# In Situ-Activated Phospholipid-Mimic Artemisinin Prodrug via Injectable Hydrogel Nano/Microsphere for Rheumatoid Arthritis Therapy

**DOI:** 10.34133/research.0003

**Published:** 2022-12-15

**Authors:** Yawei Du, Chao Li, Yu Zhang, Wei Xiong, Fei Wang, Juan Wang, Yingze Zhang, Lianfu Deng, Xinsong Li, Wei Chen, Wenguo Cui

**Affiliations:** ^1^ Department of Orthopaedics, Shanghai Key Laboratory for Prevention and Treatment of Bone and Joint Diseases, Shanghai Institute of Traumatology and Orthopaedics, Ruijin Hospital, Shanghai Jiao Tong University School of Medicine, 197 Ruijin 2nd Road, Shanghai 200025, China.; ^2^ Department of Orthopaedic Surgery, the Third Hospital of Hebei Medical University, No. 139 Ziqiang Road, Shijiazhuang 050051, China.; ^3^ School of Chemistry and Chemical Engineering, Southeast University, 2 Southeast University Road, Nanjing 211189, China.

## Abstract

In situ-activated therapy is a decent option for localized diseases with improved efficacies and reduced side effects, which is heavily dependent on the local conversion or activation of bioinert components. In this work, we applied a phospholipid-mimic artemisinin prodrug (ARP) for preparing an injectable nano/microsphere to first realize an in situ-activated therapy of the typical systemically administrated artemisinin-based medicines for a localized rheumatoid arthritis (RA) lesion. ARP is simultaneously an alternative of phospholipids and an enzyme-independent activable prodrug, which can formulate “drug-in-drug” co-delivery liposomes with cargo of partner drugs (e.g., methotrexate). To further stabilize ARP/methotrexate “drug-in-drug” liposomes (MTX/ARPL) for a long-term intra-articular retention, a liposome-embedded hydrogel nano/microsphere (MTX/ARPL@MS) was prepared. After the local injection, the MTX/ARPL could be slowly released because of imine hydrolysis and targeted to RA synovial macrophages and fibroblasts simultaneously. ARP assembly is relatively stable before cellular internalization but disassembled ARP after lysosomal escape and converted into dihydroartemisinin rapidly to realize the effective in situ activation. Taken together, phospholipid-mimic ARP was applied for the firstly localized in situ-activated RA therapy of artemisinin-based drugs, which also provided a brand-new phospholipid-mimic strategy for other systemically administrated prodrugs to realize a remodeling therapeutic schedule for localized diseases.

## Introduction

For some localized diseases, in situ-activated therapy based on the local microenvironment could be a decent option with effectively improved therapeutic effect [1,2]. Compared with systemic therapy strategy, the in situ-activated therapy can markedly reduce the side effects on normal tissues as well [2]. A typical in situ-activated therapy in local lesion mainly relies on the local conversion and activation of inert medicaments (such as prodrugs) at a particular rate to realize the interaction between medicaments and organs, target cells, or target proteins through nonspecific or specific effects, thus, to improve the disease process. Different from the direct treatment by active substances, in situ-activated therapy shows more appropriate controllability and can achieve a better long-term effect for local symptoms of chronic diseases.

As mentioned above, in situ-activated therapy usually depends on the local conversion and activation of a nonactivated component [3–5]. Prodrugs are usually the most important part in those therapeutic schedules [4,6]. However, many prodrugs are designed to be converted in liver or kidney after systemic administration [7]. Artemisinin-based drugs (ARTs), landmarked traditional Chinese medicine derivatives, are typical systemically administered agents, which could be regarded as liver-activated prodrugs of dihydroartemisinin (DHA) [8–10]. As reported, liver cytochrome P450 (CYP450) enzymes are necessary for the metabolism and conversion of ARTs [11]. However, limited by the autoinduction metabolism, ARTs always demonstrated an extremely short half-life after multiple dosing [11]. Even worse, the poor solubility of ARTs makes the increasing probability of precipitation in local physiological environment, which further limits their local administration [12]. Previously, ARTs are reasonably free from the local administration issues as antimalarial medicines. However, the situation is different while considering their increasingly found efficacies in a variety of diseases, especially some localized diseases such as tumor, inflammation, and cardiovascular diseases [13,14]. Apparently, traditional ART administration is lack of in situ activation for localized diseases, which greatly restricts their further application for these newly discovered adaptation diseases.

Rheumatoid arthritis (RA) is an autoimmune disease primarily affecting joints [15]. Currently, the RA therapy heavily relies on the systemic administration of long-term disease-modifying antirheumatic drugs (DMARDs) [16]. However, it cannot be ignored that systemically administrated DMARDs have many side effects, including gastrointestinal reaction, liver and kidney function injury, leukopenia, and thrombocytopenia [17]. In contrast to systemic administration, local administration by intra-articular injection can rapidly improve joint conditions and reduce side effects, which can build patient confidence as well [5]. Although the active drugs injected in the joint cavity can improve the condition temporarily, they could also be easily and rapidly inactivated because of the presence of local metabolic enzymes and other factors, which may not help to achieving the purpose of long-term treatment. Therefore, the abovementioned in situ-activated therapy based on local microenvironment would be a more sensible choice for a long-term effect [18]. However, there is no specific local in situ-activated therapy for RA in clinic so far. Although some inert biomaterials, such as hyaluronic acid (HA), are reported with lubrication function to relieve symptoms of arthritis, the clinical efficacies are irregular [19,20]. Considering the high hyaluronidase microenvironment in RA joint cavity that can promote the degradation of HA, crosslinked HA materials could be used as drug carriers to realize a sensitively controlled release [19,21]. In our previous works, hydrogel microspheres prepared by microfluidic technology based on photocrosslinkable methacrylated HA (HAMA) were used as drug or cell carriers for local intra-articular injection to pursue a long-term activity [19,22]. Through the combination of HAMA hydrogel microspheres and local convertible prodrugs, a precise and long-term in situ activation strategy could be realized after intra-articular injection and after achieving a disease matching treatment.

**Fig. 1.**
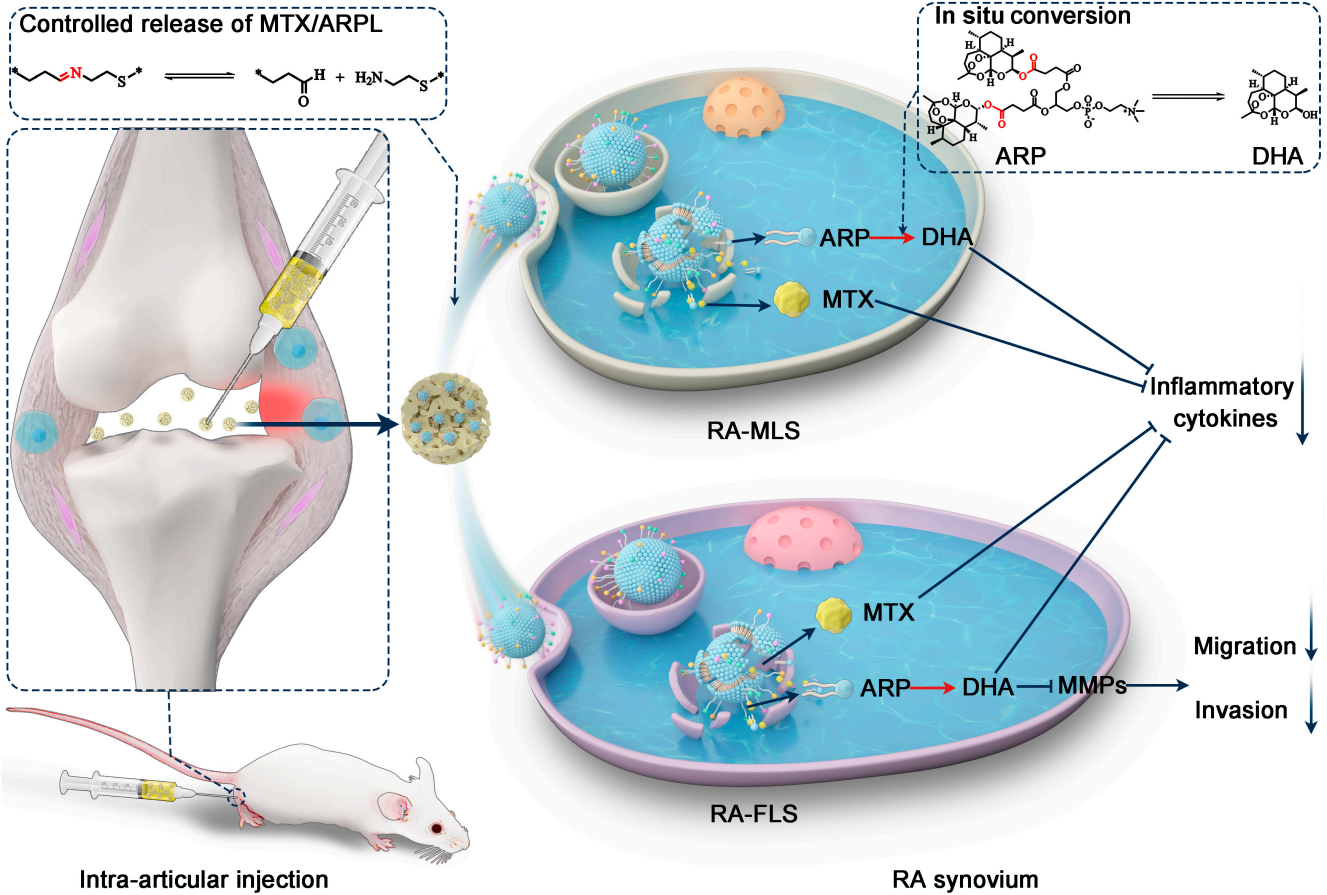
An injectable ARP hydrogel nano/microsphere was prepared for the intra-articular local injection, which was also the first time to obtain artemisinin-based in situ-activated RA therapy. After the local injection, the MTX/ARPL liposomes could be slowly released from the microspheres and targeted to the RA synovial macrophages (RA-MLS) and fibroblasts (RA-FLS). After cellular internalization, the disassembled ARP could be converted into DHA without CYP450 enzymes to realize effective in situ activation. Combination therapy of DHA and MTX would down-regulated the expression of inflammatory cytokines of both cells and inhibit the migration and invasion of RA-FLS.

In a previous work, a novel phospholipid-mimic artemisinin prodrug (ARP) was reported as a medicative alternative of traditional phospholipids to assemble liposomes directly [23,24]. However, its local delivery for in situ-activated therapy of localized diseases was never considered before the efficient CYP450-independent conversion was found, which may be attributed to the zwitterionic phospholipid head [24]. In this work, we first applied ARP for local delivery to realize an in situ-activated therapy for a localized lesion. Meanwhile, an injectable ARP hydrogel nano/microsphere was prepared for the intra-articular local injection, which was also the first time to obtain artemisinin-based in situ-activated RA therapy (Fig. 1). First, ARP was synthesized by heterogeneous esterification reaction. Then, “drug-in-drug” liposomes were prepared using medicated ARP as the major component with imine-thiol, folate (FA), and peptide SFHQFARATLAS (HAP-1) peptide surface modification to encapsulate methotrexate (MTX/ARPL). MTX/ARPL was mixed in the HAMA phase during the microfluidic microsphere preparation. The photocrosslinking would also happen between HAMA and MTX/ARPL via thiol-ene click reaction during the formulation of hydrogel and obtain MTX/ARPL-embedded HAMA nano/microsphere (MTX/ARPL@MS). ARP assembly is relatively stable before cellular internalization, but the disassembled ARP after lysosomal escape could be converted into DHA rapidly without CYP450 enzymes to realize effective in situ activation. Therefore, ARP was applied for local treatment for the first time and, thereby, obtained the first artemisinin-based medicine for local treatment of RA as an in situ-activated therapy. Moreover, we deeply believe that this phospholipid-mimic strategy also has the guiding significance for the remodeling local usage of other systemic administrated prodrugs to realize the in situ-activated therapy for localized diseases.

## Results and Discussion

### Synthesis of phospholipid-mimic ARP and PEGylated lipids

**Fig. 2.**
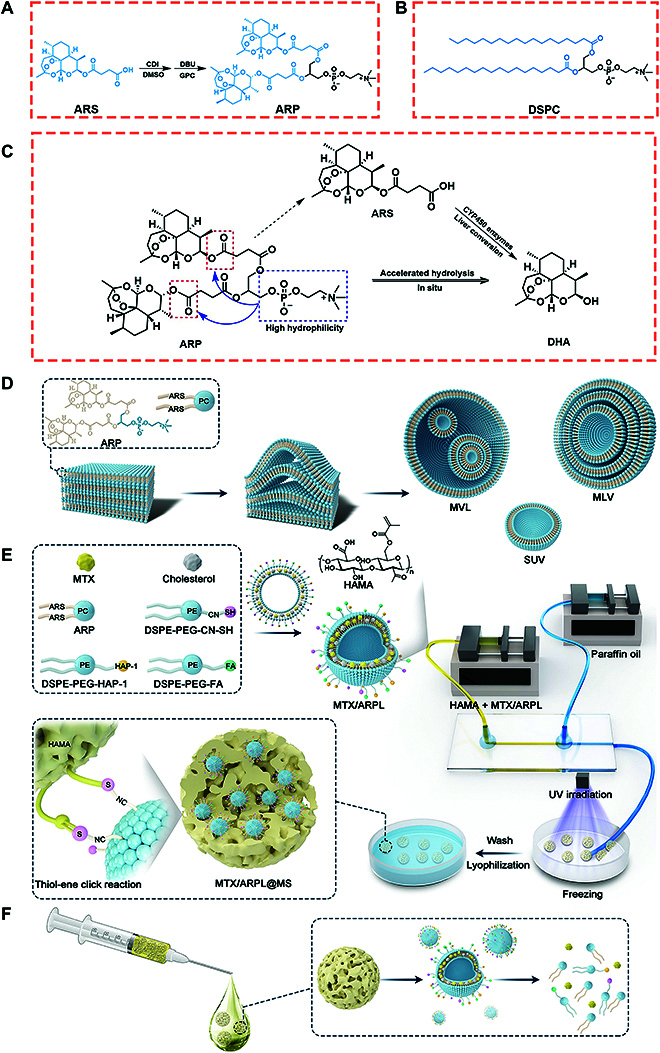
(A and B) Chemical structure of ARP (A) and 1,2-dioctadecanoyl-sn-glycero-3-phophocholine (DSPC) (B). (C) In situ conversion mechanism of ARP without participation of liver CYP450 enzymes. (D) The mechanism of ARP from thin film to different vesicle structures during thin-film dispersion method, including multivesicular liposome (MVL), multilamellar vesicle (MLV), and single unilamellar vesicle (SUV). (E) Preparation of MTX/ARPL liposome and the injectable MTX/ARPL@MS hydrogel nano/microsphere with chemical binding between liposomes and microsphere matrix. (F) The MTX/ARPL@MS hydrogel nano/microsphere is injectable, and MTX/ARPL is releasable. CDI, carbonyl diimidazole; DBU, 1,5-diazabicyclo[5.4.0]-5-undecene; DMSO, dimethyl sulfoxide.

Phospholipid-mimic ARP was synthesized by a heterogeneous esterification reaction of artesunate (ARS) and L-α-glycerylphosphorylcholine (GPC) catalyzed with carbonyl diimidazole and 1,5-diazabicyclo[5.4.0]-5-undecene (Fig. 2A and Fig. S1) [23]. ARP has a similar chemical structure with traditional phospholipids, such as 1,2-dioctadecanoyl-sn-glycero-3-phophocholine (Fig. 2B). Two ARS molecules replace aliphatic chains of sn-1 and sn-2 positions. The ^1^H-nuclear magnetic resonance (^1^H-NMR) of ARP was shown in Fig. S2; the peaks appeared as singlets at 3.3 and 1.3 parts per million (ppm) were typical methyl signals at the GPC head and ARS tails, respectively. In addition, all the other peaks were attributed to the chemical structure of ARP. The high-resolution mass spectrum (HRMS) was shown in Fig. S3; molecular ion peaks at 990.4460, 991.4498, 992.4527, and 993.4581 (charge-to-mass ratio (m/z); [M + H]^+^) were all in accordance with the calculated molecular weight of ARP (989.44, m/z) and its isotopes. All these data indicated that ARP was synthesized successfully.

Although this heterogeneous esterification reaction maybe has a relative lower yield than homogeneous reaction, the final compound could be obtained through only one step. After our continuous optimization of ARP synthesis, a stable yield higher than 50% could be achieved using carbonyl diimidazole/1,5-diazabicyclo[5.4.0]-5-undecene, which could totally satisfy further clinical transformation. Moreover, the cost is also controllable as all reagents are available with low prices, and no special equipment is needed.

According to literatures, artemisinins (ARPs) (e.g., ARS) need to be converted to active DHA metabolite for high potency, which is usually effectively catalyzed by CYP450 enzymes in liver [24]. From high-performance liquid chromatography (HPLC) (Fig. S4) and liquid chromatography-tandem mass spectrometry (LC-MS/MS) (Figs. S5 and S6) data, ARS showed slow conversion to DHA in a neutral environment lacking of esterase. By contrast, free ARP could continually be converted to DHA with a much faster rate, and only trace amount of ARS was detected, indicating that the conversion from ARP to DHA probably do not need to go through the ARS intermediate. It can be speculated that the obviously accelerated hydrolysis of the ester bond in the ARS molecules of ARP compound may be attributed to the dramatically increased hydrophilicity from the zwitterionic GPC head (Fig. 2C). Thus, the fast conversion from free ARP to active DHA is the ensurance of in situ-activated therapy in local treatment.

Although ARS is the only clinical injectable artemisinin-based drug (in 5% sodium bicarbonate), it only possesses a limited water solubility and still has a risk of precipitation in neutral physiological environment [25]. Especially for local intra-articular injection with high concentration, ARS with uncontrollable precipitation may aggravate arthritis symptoms. Therefore, a highly soluble ARP with enzyme-independent DHA conversion is a great potential artemisinin injection for local administration by in situ-activated therapy.

Except ARP, polyethylene glycol (PEG) covalently modified (PEGylated) lipids, including DSPE-PEG-CN-SH and terminal sulfydryl- or HAP-1-modified 1, 2-distearoyl-sn-glycero-3-phosphoethanolamine-PEG (named as DSPE-PEG-CN-SH and DSPE-PEG-HAP-1, respectively), were also synthesized before the liposomal preparation. Briefly, DSPE-PEG-CN-SH was synthesized by DSPE-PEG-aldehyde group (CHO) and cysteamine via Schiff base reaction (Figs. S7 and S8) [26], and DSPE-PEG-HAP-1 was synthesized by DSPE-PEG-NHS and HAP-1 peptide via amidation reaction [27], which was used for M1-type macrophage targeting [28]. DSPE-PEG-FA was directly purchased from the supplier, which was used for synovial macrophages targeting [29].

ARP is an amphiphilic prodrug that shared a similar structure with traditional phospholipids. Therefore, it could self-assemble into unique medicative vesicae without any excipients [23]. By this way, we can achieve a stable delivery system with an extremely high drug-loading capacity (>60%), which is much higher than the traditional nanostructure carriers. Using ARP as an alternative of traditional inert phospholipids, we can further obtain a “drug-in-drug” carrier system for co-delivery. Most importantly, ARP would have an efficient CYP450-independent conversion in localized lesions, which can generate an autologous in situ activation.

### Preparation of in situ activatable MTX/ARPL liposome

Owing to the similar structure with traditional phospholipids, ARP has a great assembly ability into liposomes (Fig. 2D) [24]. Different with conventional liposomes, ARP liposome (ARPL) per se is a prodrug and could be used as a medicative carrier to encapsulate a second drug for combination therapy [30]. Although we had applied the ARP and its assemblies in some disease models, ARP formulations were always acted for systemic administration [30,31]. Therefore, we focused on this specific enzyme-independent efficient local conversion of ARP for the first attempt of artemisinin-based medicine for local in situ-activated treatment of a localized disease. A locally administrated “drug-in-drug” co-delivery liposome (MTX/ARPL) was prepared by employing ARPL carrier to load MTX, which is the first choice among DMARDs for clinical RA combination therapy (Fig. 2E). Combination drug therapy of DMARDs is a clinical consensus in the RA treatment. Moreover, MTX is widely accepted to be used as the cargo for many kinds of nanodrug delivery systems including liposomes for RA treatment. ARPL is a novel liposomal carrier and shared similar physicochemical properties with traditional liposomes. Nanomedicines are widely accepted with great potential for autoimmune diseases like RA [32,33]. Thus, it is reasonable for application of MTX delivery systems because such nanocarriers could effectively avoid the systemic or gastrointestinal side effects of MTX.

**Fig. 3.**
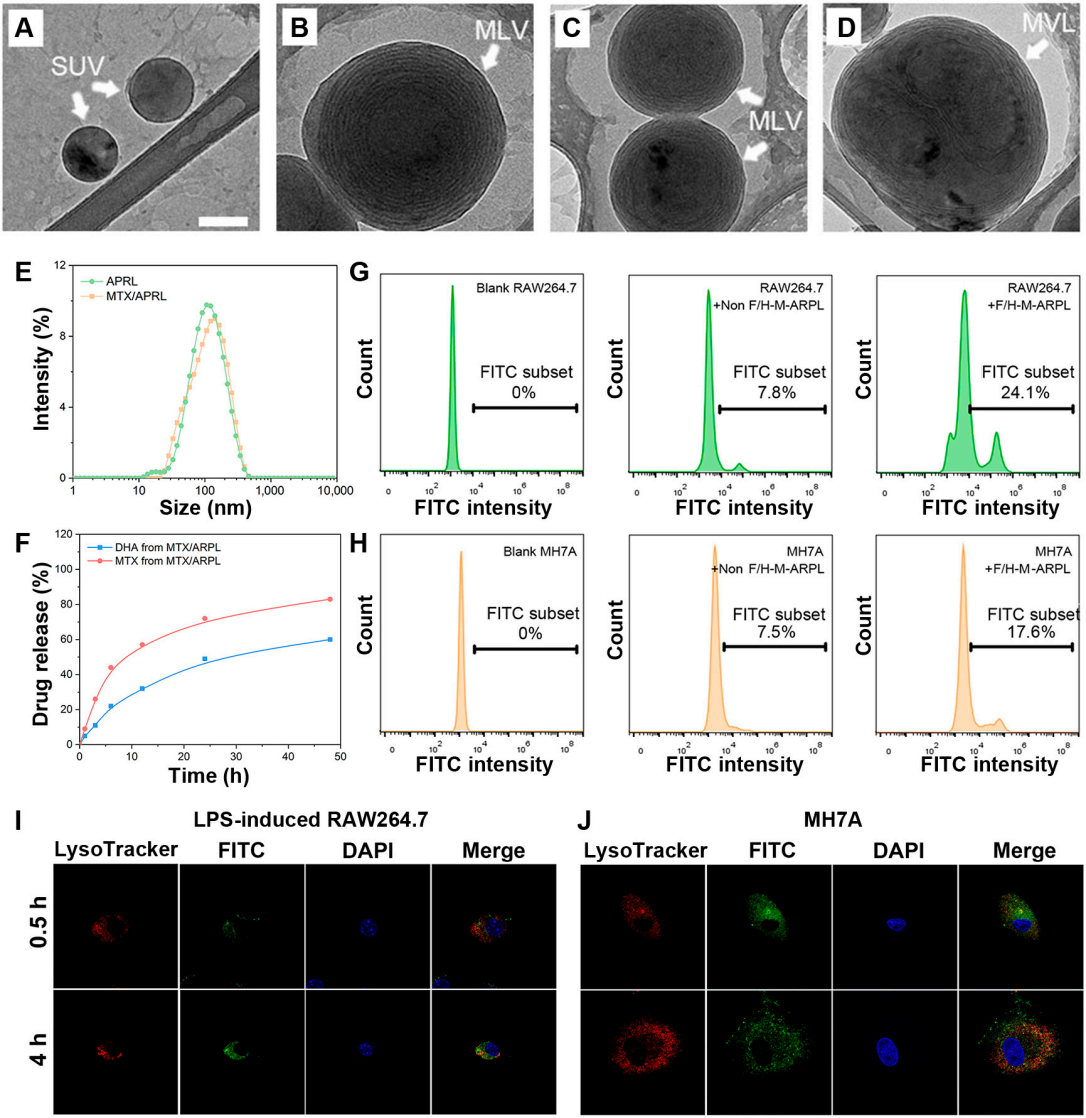
Characterization of MTX/ARPL. (A to D) CryoEM images of MTX/ARPL clearly presented liposomal vesicae with different structure, including SUV, MLV, and MVL. (E) Size distribution of ARPL and MTX/ARPL. (F) Release curves of DHA and MTX from MTX/ARPL in the simulated tissue fluid. (G and H) Cellular uptake behavior of liposomes without FA and HAP-1 modifications (Non F/H-M-ARPL) and liposomes with modifications (F/H-M-ARPL) toward LPS-induced RAW264.7 cells (G) and MH7A cells (H). (I and J) Lysosomal escape assay of MTX/ARPL against LPS-induced RAW264.7 cells (I) and MH7A cells (J).

Through classic thin-film dispersion method, MTX/ARPL was prepared by ARP, cholesterol, and PEGylated lipids [34]. Under the particular ratio (see the Supplementary Materials and Methods section), the drug loading rate and encapsulation efficiency were 9.5 ± 0.1% and 98.6 ± 0.9%, respectively. The morphology of liposomes was observed by cryo-electron microscopy (CryoEM). As shown in Fig. S9A to D, the CryoEM images of self-assembled ARPL without MTX loading clearly presented liposomal vesicae with different structures, including single unilamellar vesicles (SUV), large unilamellar vesicles (LUV), multilamellar vesicles (MLV), and multivesicular liposomes (MVL), which were prepared according the previously published method [24]. After MTX loading, the different liposomal vesicae were still recognizable, but the contrast was obviously different with ARPL, which should be attributed to the MTX (Fig. 3A to D). Moreover, the slightly increased size distribution of MTX/ARPL was observed, which was also reflected in the dynamic light scattering-acquired data (Fig. 3E). The MTX encapsulation could slightly affect the size distribution, and the average size of ARPL and MTX/ARPL were 168 ± 13 nm and 184 ± 9 nm, respectively.

We assumed that a multilamellar structure would be beneficial for the stability of ARPL. In general, the multilayered structure is always regarded as the fact contributing to the stability of vesicae [35,36]. Considering the fast conversion from free ARP to DHA, the multilamellar core–shell structure is necessary to protect the ARPL from uncontrollable rapid activation during storage before injection. Therefore, the multilamellar structure is preferred for ARPL. Although multilamellar structure will result in a slightly larger size distribution, it is not a big deal for the liposomes used in the local sites by direct injection. In contrast, liposomes with smaller size, especially SUV, are still the best option for systemic injection, as we discussed before [31].

The MTX and converted DHA released from MTX/ARPL were measured by ultraviolet (UV) spectroscopy and LC-MS, respectively. As shown in Fig. 3F, DHA and MTX could be released from MTX/ARPL effectively. After incubation for 24 h, the accumulated MTX and DHA release rates were 70% and 45%. After that, the release rate tended to slow, and the accumulated MTX and DHA release rates reached approximately 80% and 55%, respectively. Although MTX and DHA experienced similar release behavior, their release routes are totally different. Because MTX is just the cargo of ARPL, the release of MTX is mostly a physical process, and the degradation of phospholipids will accelerate the process. Meanwhile, the release of DHA is a chemical process that refers to the hydrolysis of ester bonds. As mentioned above, the ARP could be converted to DHA in a neutral medium without any esterase, which is different with other ARPs like ARS, artemether, and arteether. Although ARP is a derivative of ARS, the conversion rate of ARP is far faster than ARS (Fig. S6). It may be attributed by enhanced water-solubility, which expedites the hydrolysis rate. It is assumed that the disassembly and conversion could be accelerated if ARPL enters a weakly acidic lysosome environment after endocytosis. From the release assay, the degradation of ARP and the release rate of cargo are relatively fast when compared with traditional liposomes. It means that the merely free ARPL or MTX/ARPL could not be used directly in local long-term treatment. Therefore, a further nano/microsphere system would be adopted to immobilize ARP-based liposomes. With the protection of the formulated lipid bilayer and microsphere structure, the sensitive artemisinin peroxide bridge in ARP hydrophobic tail could be stabilized [37]. Once the ARPL was released and internalized by cells, the disassembled free ARP could be converted into active DHA effectively.

### Cellular uptake of MTX/ARPL liposome

The cellular uptake behavior of MTX/ARPL was studied by flow cytometry. Two ligands (FA and HAP-1 peptide) were applied for the surface modification of MTX/ARPL to target inflammatory macrophage and synovial fibroblast according to the literatures [28,29]. In addition, DSPE-PEG-fluorescein isothiocyanate (FITC) was added in the liposomal formulation to label MTX/ARPL with fluorescence signal. Lipopolysaccharide (LPS) (and interferon-γ (IFN-γ)) induced RAW264.7 macrophages (M1-type), and RA synovial fibroblasts (MH7A) were incubated with FITC-labeled MTX/ARPL for 2 h [38]. Then, the intracellular FITC intensity was analyzed using a flow cytometer. As shown in Fig. 3G and H, both LPS-induced RAW264.7 and MH7A cells demonstrated relatively high intracellular FITC intensity when incubated with FA/HAP-1-modified MTX/ARPL (F/H-M-ARPL), when compared with the same cells incubated with non-modified MTX/ARPL (Non F/H-M-ARPL). In LPS-induced RAW264.7 cells, the FITC-positive rate of the F/H-M-ARPL group was 24.1%, while the value of the Non F/H-M-ARPL group was only 7.8%. Similarly, the F/H-M-ARPL and Non F/H-M-ARPL groups also showed marked difference in MH7A cells, and the corresponding FITC-positive rates were 17.6% and 7.5%, respectively. These data revealed that FA and HAP-1 peptides could effectively improve the cellular uptake of MTX/ARPL into LPS-induced RAW264.7 and MH7A cells.

Moreover, a lysosomal escape assay of MTX/ARPL against macrophages and fibroblasts was carried out by laser scanning confocal microscope via lysosome and liposome colocation [39]. The lysosomes were stained by LysoTracker Red, and liposomes were labeled by co-encapsulation with an FITC dye. As shown in Fig. 3I and J, the FITC and LysoTracker Red fluorescence were only partially overlapped after being internalized for 0.5 h in both LPS-induced RAW264.7 macrophages and MH7A fibroblasts. After 4 h, the FITC signals dispersed in the whole cytoplasm rather than the lysosomal location. From these data, a slight lysosomal escape of MTX/ARPL occurred merely after cellular uptake for 0.5 h, and a much marked escape happened after a longer incubation.

In general, most nanoparticles will be internalized by endocytosis via lysosomes. A marked overlap of staining of lysosomes and nanoparticles would be observed under a fluorescence microscope. On the basis of this, we designed this colocation experiment to figure out the uptake pathway of ARPL against cells. In our previous understanding, ARPL would be internalized by lysosome-mediated endocytosis, which would reflect in the highly overlapping fluorescence from ARPL and lysosome. Basically, the consistency of two fluorescence signals would be noticed, demonstrating that endocytosis should be the main uptake way of ARPL. However, the fluorescence signals of lysosomes and liposomes did not overlap completely after 0.5 h of incubation. Thus, we suspected that other approaches such as membrane fusion may also attribute to the cellular uptake of ARPL.

### Preparation of injectable MTX/ARPL@MS hydrogel nano/microsphere

The local administration of HA is commonly acceptable in the clinical RA treatment. Here, an HA-based microsphere with internal highly crosslinked structure was used to further stabilize MTX/ARPL for long-term local administration. Microfluidic technique was used for the preparation of MTX/ARPL-HAMA nano/microspheres (MTX/ARPL@MS). Similar photocrosslinked HAMA hydrogel microspheres were well studied in our previous works [19,22,40,41]. Intra-articular injection of HAMA hydrogel microspheres can improve the lubricity of joint cavity to ease arthritis symptoms.

**Fig. 4.**
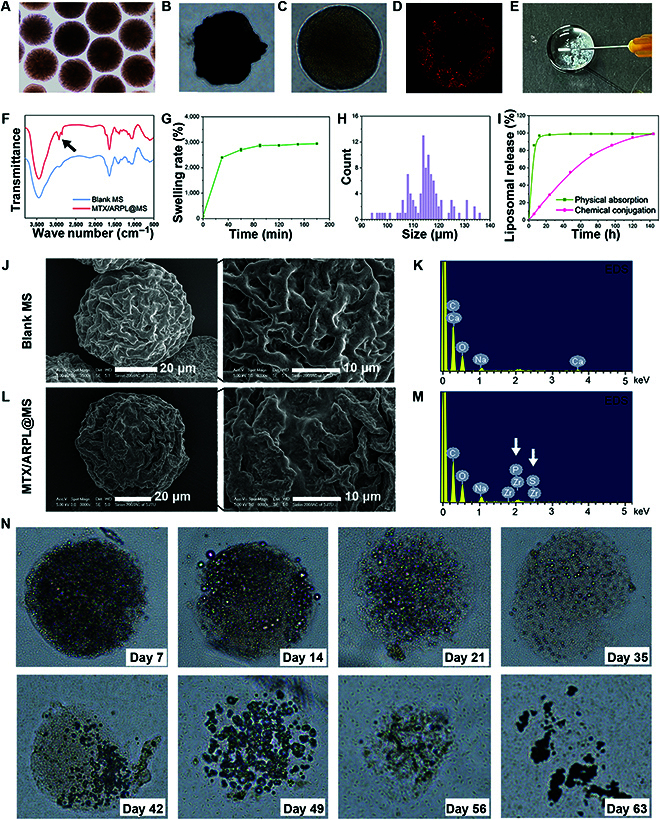
Characterization of MTX/ARPL@MS. (A to C) Optical microscope images of MTX/ARPL@MS before (A) and after (B) lyophilization and after reswell (C). (D) Fluorescence microscope image showing the location of MTX/ARPL in the microsphere after DiR labeling. (E) The injectability of MTX/ARPL@MS. (F) Fourier transform infrared spectrum of blank microsphere (Blank MS) and MTX/ARPL@MS. (G) Swelling rate of lyophilized MTX/ARPL@MS. (H) Size distribution of MTX/ARPL@MS according to optical microscope images. (I) Liposomal release curves of nonconjugated loading (physical absorption) and thiol-ene click reaction-based loading (chemical conjugation). (J to M) Transmission electron microscopy and EDS of blank microsphere (Blank MS) (J and K) and MTX/ARPL@MS (L and M). (N) Optical microscope images of degraded MTX/ARPL@MS after different incubation times in the simulated tissue fluid.

Porous MTX/ARPL@MS was firstly prepared by microfluidic technique (as shown in Fig. 2E). To effectively immobilize nanosized MTX/ARPL in the porous HAMA hydrogel microspheres, thiol was induced by adding DSPE-PEG-CN-SH in the liposomal formulation, which could conjugate with HAMA simultaneously by thiol-ene click reaction while the photoinitiated crosslink happens [42]. Beneficial to the reversible hydrolysis of the Schiff base imine bond, MTX/ARPL could be released from the porous nano/microspheres. Before the lyophilization, nano/microspheres with uniform particle size could be observed using an optical microscope (Fig. 4A). Shrinkage happened after lyophilization, and the nano/microsphere was presented with an irregular shape (Fig. 4B). However, the shape of MTX/ARPL@MS could be restored once it was immerged into the aqueous phase (Fig. 4C). With 1,1-dioctadecyl-3,3,3,3-tetramethylindotricarbocyanine iodide (DiR) labeling, the location of MTX/ARPL could be observed using a fluorescence microscope clearly (Fig. 4D). The fluorescence of liposomes appears, surrounding the outer sphere of the microsphere. We assumed that this should be a common phenomenon for microspheres because of the different thicknesses on the different points of the same cross section. In addition, the loading of MTX/ARPL was also reflected in the Fourier transform infrared spectrum, in which the absorption peaks (indicated by an arrow) at around 2800 cm^−1^ may be attributed to the stretching vibration-saturated carbons of phospholipid-mimic ARP and PEG chains (Fig. 4F). The recuperative MTX/ARPL@MS could go through a syringe needle easily, which demonstrate their injectability (Fig. 4E). During the water-absorbing process of nano/microsphere recuperation, the wet weight of MTX/ARPL@MS was recorded and calculated as the swelling rate, as shown in Fig. 4G. The swelling mostly happened within the first 40 min and finally reached an equilibrium swelling rate of around 3000%. Moreover, the size of the recuperative MTX/ARPL@MS was measured using an optical microscope.

The size distribution was shown in Fig. 4H, and the calculated average value was 115 ± 8 μm. The morphology of a blank microsphere (Blank MS) and MTX/ARPL@MS was observed using a scanning electron microscope, and the chemical element information was analyzed using an energy-dispersive spectrometer (EDS) simultaneously. As shown in Fig. 4J and L), a plicated porous structure was observed in both of Blank MS and MTX/ARPL@MS samples, while the ravine on the surface of MTX/ARPL@MS was relatively smooth compared with the sample of Blank MS. The shrunken lyophilized microspheres showed a much smaller size (with diameters of around 40 μm) compared the aquiferous microspheres (under an optical microscope). Moreover, the EDS data clearly showed the emergence of S and P elements, which further confirmed the of doping of MTX/ARPL liposomes (Fig. 4K and M).

Under the UV irradiation, MTX/ARPL formed thioether covalent bonds with HAMA matrix in the microspheres and was then slowly released through the hydrolysis of Schiff base imine bonds [42]. To investigate the effect of this prolonged release mechanism, the liposomal release behaviors of nano/microspheres with noncovalent (physical absorption) and covalent combination (chemical conjugation) were detected by a UV spectrum. MTX/ARPL@MS spheres were immerged in a simulated joint fluid containing hyaluronidase. As shown in Fig. 4I, the nano/microspheres with covalent combination (chemical conjugation) obviously had the flatter release curve than noncovalent microspheres. The saturated release time of the covalent combination was around 6 d, while the noncovalent combination released 85% within 6 h and got saturated release only after 12 h. Apparently, the imine bond/thioether conjunction will markedly immobilize MTX/ARPL in the HAMA microspheres to prevent rapid release.

In the same simulated joint fluid condition, the degradation of MTX/ARPL@MS was further observed the profile and morphology using an optical microscope. The photos were shown in Fig. 4N. In the first 7 d, only partial surface degradation of the microspheres occurred. Starting from day 14, the main structure of the microspheres began to disintegrate. Finally, the microsphere structure becomes completely invisible after about 9 wk. It is quite clear that HAMA microspheres can be effectively degraded in the simulated joint fluid condition. Within the first 7 d, the major structure of microspheres was retained while the MTX/ARPL was released sustainably. After 7 d, almost all MTX/ARPL were released and just left the HAMA microspheres, and their major structure was also markedly destroyed. Considering the lubricant function of HA, the blank spheres and degraded HA molecules still have additional positive function to the joint wear situation, which can continuously improve the joint comfort of patients.

HAMA hydrogel microsphere is very important in this nano/microsystem, which could be explained from two perspectives. First, HA pe ser has considerable lubrication properties, which could be used directly as intra-articular injections in the clinic treatment. In addition, microspheres based on hydrophilic polymers could also be attributed to the lubrication in the articular cavity because of their ball-bearing-like sharp property [43]. Second, the ARPL would be internalized rapidly by immune cells and synovioblasts without the protection of microspheres. In addition, the cargo and DHA would be released slightly quicker from free ARPL compared with the nano/microsystem. That would be unfriendly to the long-term RA treatment. Therefore, we believe that this nano/microsphere structure is necessary.

### In vitro cytotoxicity of injectable MTX/ARPL@MS hydrogel nano/microsphere

**Fig. 5.**
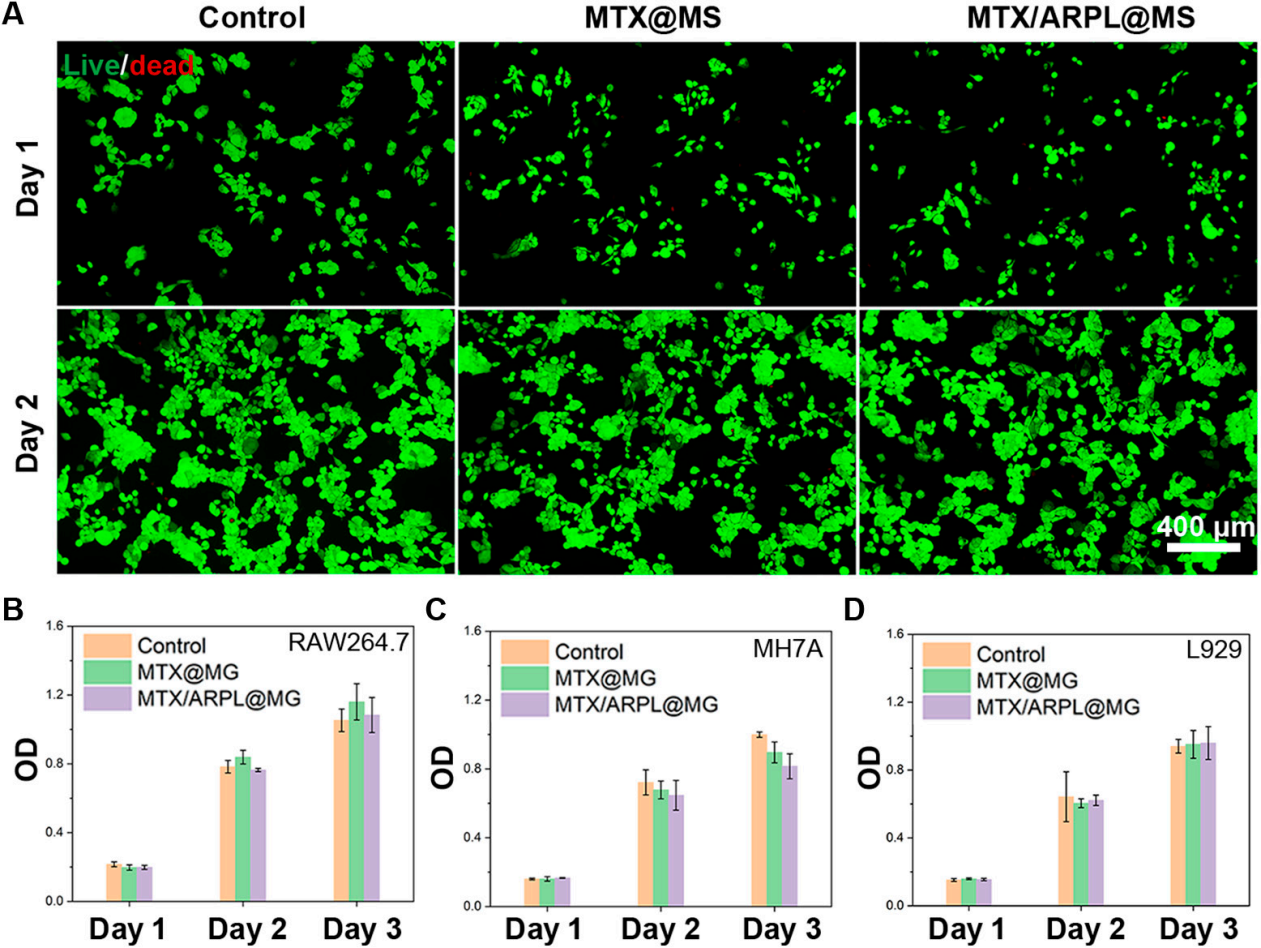
In vitro cytotoxicity assay of MTX/ARPL@MS. (A) Live/dead analysis of MH7A cells after different treatments via calcein-AM/PI staining. (B to D) CCK8 assay to detect the cell viabilities of RAW264.7, MH7A, and L929 cell lines after different treatments for 1 to 3 d.

The in vitro cytotoxicity of MTX/ARPL@MS was evaluated by a live/dead cell situation via calcein acetoxymethyl ester/propidium iodide (calcein–AM/PI) double staining and viability measurement via Cell Counting Kit-8 (CCK8) assay. For live/dead staining assay, human RA synovial (MH7A) cells were incubated with MTX liposome/microspheres (MTX@MS) and MTX/ARPL@MS for 1 or 2 d and then stained by a calcein–AM/PI kit. The images were shown in Fig. 5A; the live and dead cells were stained with green and red fluorescence, respectively. From the images, almost all cells observed in each group at two time points were alive, which were stained by green fluorescence, and very few cells showed red fluorescence. In addition, no marked difference was observed in different groups, indicating that both microspheres were safe.

Then, three different cell lines were used to further assess the cytotoxicity of the microspheres by CCK8 assay, including mouse mononuclear macrophage (RAW264.7), MH7A, and mouse fibroblast (L929). Cells were incubated with MTX@MS and MTX/ARPL@MS for 1 to 3 d, and then the cell viabilities were evaluated by a colorimetric method after adding CCK8. The cell viability data were shown in Fig. 5B to D. The positive time dependence of optical density (OD) values was found in all cell lines, which means that cell proliferation did not be inhibited after incubation with microspheres. In general, there was no marked difference among all groups including the blank control, especially for RAW264.7 and L929 cell lines. However, a slight inhibition of cell proliferation of MTX@MS and MTX/ARPL@MS against MH7A cells was observed (*P* > 0.05), and MTX/ARPL@MS even had a bit stronger effect than MTX@MS (*P* > 0.05). Probably, this inhibition was attributed to the pharmaceutical effect of MTX or DHA. According to literatures, MTX and artemisinin-based drugs had inhibition for the proliferation of RA synovial cells, which would beneficial to relieve abnormal synovial hyperplasia.

Through the in vitro cytotoxicity assay, the biosafety of MTX/ARPL@MS was preliminarily verified. The toxicity of ARP and ARPL was well-studied in our previous works, which showed an even higher half-lethal dose than ARS [23]. Artemisinins, including ARS, themselves are regarded as quite safe drugs, and few serious adverse reactions have been observed in a large number of clinical antimalarial treatments. However, although the probability is relatively rare, artemisinins are still reported with some potential neurotoxicity, damage to liver and kidney function, and possible reticulocenia [44]. The other drug, MTX, is reported with more adverse reactions—long-term use will lead to liver and kidney function damage, leukocyte thrombocytopenia, and infant development malformations [17]. Here, the liposomal structure and HAMA hydrogel microspheres, or nano/microsphere system, provided a release rate change for immobilized drugs, which could extremely reduce the toxicity of drugs. The safety of HAMA hydrogel microspheres has also been reported many times [20,21]. HA itself has a very low immunogenicity, and the degradation products are also very safe [45].

### In vitro anti-RA assay via analyzing inflammatory expression of M1-type macrophages

**Fig. 6.**
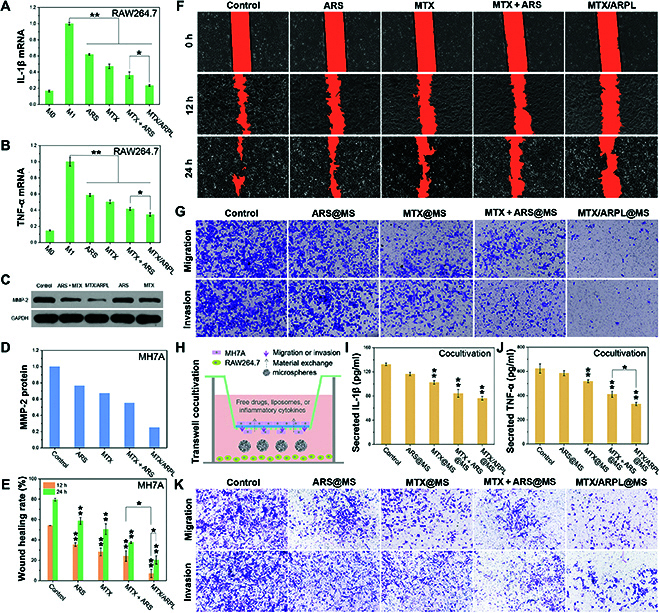
In vitro anti-RA assay. (A and B) IL-1β (A) and TNF-α (B) mRNA relative expression levels of LPS-induced (or not) RAW264.7 cells after different treatment via quantitative polymerase chain reaction analysis. (C and D) The blots (C) and semiquantitative analysis (D) of MMP-2 protein expression in MH7A cells after different treatment via Western blot. (E and F) Scratched wound images (F) and semiquantitative analysis (E) of wound healing migration assay of MH7A cells after different treatments. (G) Migration and invasion of MH7A cells after different treatment via Transwell assay. (H) Illustration of LPS-induced RAW264.7 and MH7A cocultivation. (I and J) IL-1β (I) and TNF-α (J) cytokine expression levels of LPS-induced RAW264.7 cells after different treatment during cocultivation via ELISA analysis. (K) Migration and invasion of MH7A cells after different treatment during cocultivation via Transwell assay (*P < 0.05 and **P < 0.01).

Nonactivated monocytes (M0-type) can be induced to polarize into proinflammatory (M1-type) macrophages under the stimulation of IFN-γ and LPS and secrete a large number of proinflammatory cytokines, such as interleukin-1 (IL-1), IL-6, tumor necrosis factor–α (TNF-α), and inducible nitric oxide synthase [27]. Synovial M1-type macrophages play a crucial role in the pathogenesis of RA and other diseases. Reducing the inflammatory expression of synovial macrophages will effectively alleviate the acute symptoms of RA [31]. During this assay, the inflammatory inhibitory effect of MTX/ARPL against M1-polarized macrophages was evaluated by quantitative polymerase chain reaction via detecting the mRNA levels of IL-1β and TNF-α. First, RAW264.7 cells (M0-type) were induced into M1 type by LPS and IFN-γ. Compared with M0-type macrophages, M1-type macrophages expressed markedly increased inflammatory cytokines as shown in Fig. 6A and B. After incubation with ARS, MTX, MTX + ARS, and MTX/ARPL, the mRNA levels of IL-1β and TNF-α expressed in M1-type macrophages were significantly decreased (*P* < 0.01), and the levels in MTX/ARPL group were closest to those in the M0-type control group. These indicated that MTX/ARPL had the strongest ability to inhibit the inflammatory expression of M1-type macrophages. The inflammatory inhibition of ARS and MTX on M1-type macrophages has been widely reported [46,47]. Meanwhile, the combination of ARS and MTX also showed further improved efficacy as reported [48]. Compared with MTX + ARS, the significantly higher anti-inflammatory effect of MTX/ARPL (*P* < 0.05) may be due to its unique “drug-in-drug” coassembly structure. Moreover, both water-soluble negatively charged ARS and water-insoluble MTX have relatively limited cellular uptake, but MTX/ARPL can overcome this defect well.

The inhibitory effect of artemisinins on macrophage inflammatory cytokines has been widely reported [49]. In the in vivo inflammatory environment, the M1/M2 balance would incline to M1, and more excretive proinflammatory cytokines would recruit more macrophages and induce high matrix metalloproteinases (MMPs), which may further affect the proliferation and invasion of synovial fibroblasts [49]. Thereby, inhibiting the polarization of M1 type and the secretion of inflammatory cytokines is one of the main strategies to treat RA. Large numbers of literatures have reported that artemisinins can markedly reduce the expression of inflammatory factors in M1-polarized macrophages, especially TNF-α [50]. The main mechanism should be related to the expression of Toll-like receptor 2 (TLR) and the inhibition of nuclear factor κB (NF-κB) activation [51]. Moreover, artemisinins can down-regulate the production of NO, which is also an important immunomodulatory mechanism of inflammation [52]. As a new artemisinin derivative, ARP has much higher stability and solubility than traditional artemisinin. At the same time, it has excellent self-assembly ability and accompanying improved stability. Once taken by M1-polarized macrophages, ARP would be converted to active DHA rapidly for in situ-activated therapy.

### In vitro anti-RA assay via analyzing migration/invasion of RA-FLS

In addition to macrophages (or macrophage-like synoviocytes, MLS), synovial fibroblasts (or fibroblast-like synoviocytes, FLS) are another important component of the synovial membrane [53]. In RA disease, synovial fibroblasts (RA-FLS) show abnormal activation and proliferation, which are the main effector cells mediating joint destruction and synovial inflammation. The MMP-2 protein is considered to be highly involved in cell migration and invasion because it can cleave extracellular matrix components, which also has close connection with the inflammatory cytokines from RA-MLS or RA-FLS [54]. The expression of MMP-2 in human RA-FLS (MH7A) stimulated by different drugs was first detected by Western blot. As shown in Fig. 6C, the MTX/ARPL group showed markedly weaker bands than the other groups. By semiquantitative analysis, the expression of MMP-2 in MH7A cells incubated with MTX/ARPL was only one-fifth of that in untreated MH7A cells and one-half of that in the MTX + ARS group (Fig. 6D). It can be inferred that the strong inhibitory effect of MTX/ARPL on MMP-2 should have a positive effect on reducing the migration and invasion of RA-FLS.

To confirm this hypothesis, the migration inhibition effect of MTX/ARPL on MH7A cells was evaluated by cell scratch wound healing assay. The data were shown in Fig. 6E and F, and the scratched gap is marked in orange color to facilitate the semiquantitative analysis. The semiquantitative data were presented in Fig. 6E. Obviously, the wound healing of the MTX/ARPL group was significantly slower compared with other groups (*P* < 0.01), and the healing rates after 12 and 24 h were only 7.1 ± 4.1% and 20.4 ± 4.1%, respectively. After 12 h, the healing rates of control, ARS, MTX, and MTX + ARS groups were 54.1 ± 0%, 35.3 ± 1.8%, 28.3 ± 3.7%, and 24.2 ± 5.3%, respectively. After 24 h, the healing rates of the corresponding groups increased to 79.5 ± 1.3%, 58.7 ± 3.4%, 50.6 ± 5.3%, and 27.7 ± 1.0%, respectively. The inhibition of MH7A cell migration by MTX/ARPL was clearly observed by the preliminary scratch wound healing assay.

The migration and invasion inhibitory effect of MTX/ARPL@MS nano/microsystem against MH7A cells was further characterized by a Transwell experiment. MH7A cells were incubated on top of the filter membrane, and the bottom wells were filled with medium containing microspheres. The pore size of the filter membrane was 8 μm. In addition, the polyester membrane was covered with Matrigel for the evaluation of cell invasion, but not for the evaluation of cell migration. After incubation for 24 h, the cells on the underside of the membrane were fixed, stained with trypan blue, and observed using an optical microscope, and the results were shown in Fig. 6G. The migration/invasion inhibition of MH7A cells in the MTX/ARPL@MS group was the most obvious, and the effect was markedly stronger than that in the MTX + ARS@MS group. These results were also consistent with the highly effective inhibition shown by MTX/ARPL in the scratch test. In contrast, the migration/invasion inhibition of MH7A cells in the ARS@MS group was lower than that in the other experimental groups, which may be due to the lack of the corresponding in situ activation mechanism of ARS in vitro. This also explains the comparable results between the MTX@MS group and the MTX + ARS@MS group. Combined with the detection results of MMP-2, it can be inferred that the inhibitory effect of drugs on migration and invasion of MH7A cells is negatively correlated with the expression of MMP-2, which is consistent with the situation reported in the literatures [48].

The abnormal proliferation and invasion of RA-FLS is one of the main manifestations of synovitis [54]. Previous studies have shown that artemisinin drugs have obvious inhibitory effects on proliferation and invasion of RA-FLS, and a variety of signaling pathways may be involved in this process [55]. Artemisinins can reduce the production of proinflammatory cytokines by inhibiting the activation of key molecules of the NF-κB pathway and the mitogen-activated protein kinase pathway, then down-regulating the level of activated MMPs, and finally inhibiting the migration and invasion of RA-FLS. In addition, the inhibition of the phosphatidylinositol-3 kinase/proteinkinase B pathway may also lead to the reduced proliferation and differentiation of RA-FLS [55]. There is no doubt that ARP, as a precursor of artemisinin, can be metabolized in vivo to produce DHA active monomer to inhibit the proliferation and invasion of RA-FLS. At the same time, it also serves as a drug carrier to further load MTX to enhance the synergistic effect, which is a widely accepted ability to inhibit the proliferation of RA-FLS by inhibiting the receptor activator of the NF-κB ligand (RANKL) [56].

### In vitro anti-RA assay via M1-type macrophages and RA-FLS cocultivation

Synovial macrophages (MLS) and synovial fibroblasts (FLS) are two prominent cellular components in synovial tissue [53]. In the microenvironment of RA lesions, RA-MLS will show more M1-type polarization and release corresponding inflammatory cytokines, which will also affect the related physiological behaviors of RA-FLS [53]. Therefore, it is necessary to comprehensively assess the anti-inflammatory effect of MTX/ARPL@MS by introducing a RA-MLS and RA-FLS cocultivation condition to maximally mimic the in vivo RA condition.

The M1-type macrophages/RA-FLS cocultivation Transwell assay was designed, and the secreted TNF-α and IL-1β from M1-type macrophages and RA-FLS as well as the migration and invasion of RA-FLS were detected simultaneously. As shown in Fig. 6H, MH7A cells were incubated in the chamber, and LPS-induced RAW264.7 cells were incubated in the bottom, which also contained a medium with microspheres. After incubation for 24 h, the medium in each well was collected for detection of IL-1β and TNF-α by enzyme-linked immunosorbent assay (ELISA), and the results were shown in Fig. 6I and J, respectively. After incubated with MTX/ARPL@MS, the total secretion of IL-1β and TNF-α in the media were 76.4 ± 2.9 and 330.3 ± 13.8 pg/ml, respectively, which were much lower than the blank control group (*P* < 0.01). Compared with MTX + ARS@MS, MTX/ARPL@MS also showed a significantly decreased TNF-α secretion (*P* < 0.05). Meanwhile, no significant difference was found between ARS@MS and control groups (*P* > 0.05). The cells on the underside of the membrane were fixed, stained with trypan blue, and observed by light microscopy, and the results were shown in Fig. 6K. Similarly, the MTX/ARPL@MS group showed the best inhibition of migration and invasion for MH7A. From these data, the high efficacy of in situ activable ARP was further confirmed, while the ARS-related groups (ARS@MS and MTX + ARS@MS) were demonstrated with higher inflammatory cytokine secretion, as well as cellular migration and invasion.

For mechanism, the released liposomal co-delivery system from microspheres would affect both RA-MLS and RA-FLS and regulate their cytokine secretion. As the complex cytokine interactions between RA-MLS and RA-FLS, this dual-targeting strategy should be more reasonable compared with acting on one type of cells solely. Obviously, migration and invasion would be stimulated principally by the up-regulated MMPs via cytokines (e.g., TNF-α and IL-1β). Moreover, artemisinins may inhibit MMPs by a more direct pathway as well. Therefore, the migration and invasion of MH7A cells in this assay could be a combined effect of multifactors.

Undoubtedly, there is an obvious mutual influence between RA-MLS and RA-FLS [53]. The pathological basis of RA is synovitis, which is mainly characterized by abnormal synovial hyperplasia. As reported, it is highly associated with the inflammatory activation of RA-FLS, demonstrating obvious proliferative features and invasiveness [53]. The secreted proinflammatory cytokines would further recruit inflammatory cells and inhibit their apoptosis, which leads to a worse inflammatory issue [53]. In this process, the overexpression of inflammatory cytokines may also be the inducement of synovial macrophage lesions, which may involve multiple mechanisms such as mitogen-activated protein kinase pathway [57]. Moreover, IL-1β–NF-κB axis is also considered to be a key pathway of RA pathogenesis [58]. Multiple interactions between different cells and cytokines will further aggravate the inflammation of RA and produce a cascade of proliferative effects. Therefore, ideal RA drugs should simultaneously target multiple cellular targets and inhibit inflammatory lesions separately. MTX, as the most commonly used long-acting drug for RA, has been intensively studied in related fields, and its efficacy on different cells, such as RA-MLS and RA-FLS, was also studied comprehensively [59].

We also realized that ARP with specific in situ conversion capacity had positive effects on RA-MLS and RA-FLS. More importantly, the novel prodrug vector based on ARP is very suitable to be attempted to further load MTX for the dual-targeted local treatment against RA-MLS and RA-FLS simultaneously. The improved activity of MTX/ARPL@MS compared with MTX + ARS@MS should be attributed to the fast in situ activation mechanism of ARP and the special “drug-in-drug” co-delivery system. ARS and MTX liposomes in MTX + ARS@MS should have similar cellular uptake with MTX/ARPL, but the more rapid in situ conversion of ARP would be beneficial for a faster lysosomal escape of liposomes and for accompanying an easier MTX release as well as a quicker DHA generation. The improved efficacy of MTX/ARPL@MS indicated a great potential for the in situ-activated therapy in vivo.

### In vivo anti-RA assay for in situ-activated therapy of RA

**Fig. 7.**
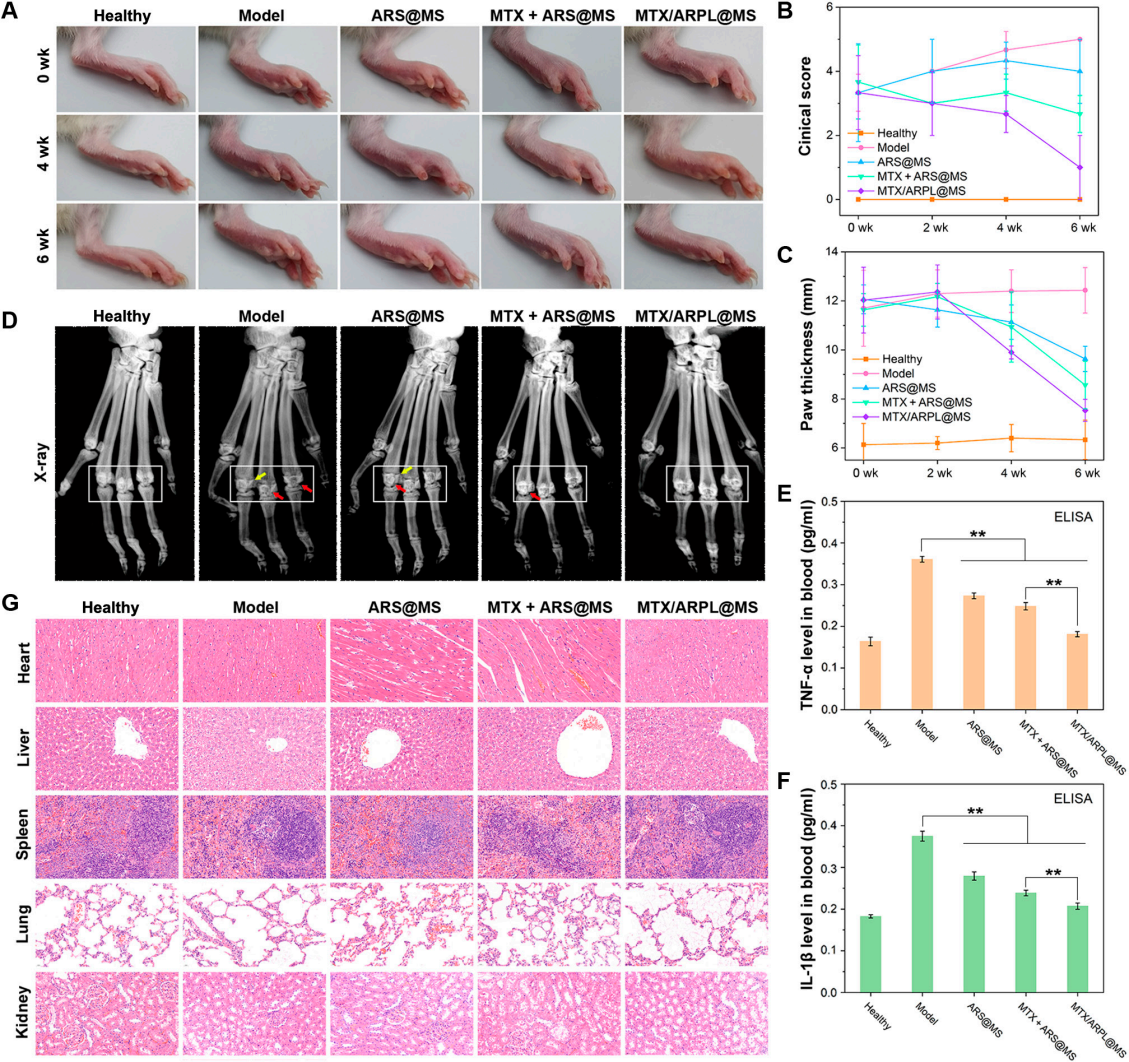
In vivo anti-RA assay. (A) Representative rat paw photos in each group after AIA modeling for 0, 4, and 6 weeks. (B) Clinical scores of rats in each group. (C) Paw thickness values of rats in each group. (D) Representative x-ray images of metatarsophalangeal joints in each group after modeling for 6 weeks. (E and F) TNF-α (R) and IL-1β (F) cytokine levels in serum samples after modeling for 6 weeks, detected by ELISA analysis. (G) H&E staining of the heart, liver, spleen, lung, and kidney tissue samples after modeling for 6 weeks (**P < 0.01).

The in vivo anti-RA efficacy assay of MTX/ARPL@MS was further evaluated by the rat adjuvant-induced arthritis (AIA) model, which could simulate the pathological changes of RA to a large extent [60]. In this study, a special two-shot AIA model was established by injecting Freund’s complete adjuvant twice a week in the paws of rats to obtain an improved effectiveness due to the enhanced secondary immunization [31]. Marked swelling, inflammation, and limitation of joint motion were observed on the whole foot especially the ankle and phalangeal joints. As reported in the literature, RA often causes swelling and pain in the distal facet joints [61]. Two weeks after modeling, injections including saline, ARS@MS, MTX + ARS@MS, and MTX/ARPL@MS started to be administrated by intra-articular injection into ankle joints. The injection was applied once a week. After injection for 4 and 6 weeks, photos of paws were taken, as shown in Fig. 7A, and the clinical scores and paw thickness values of rats were also recorded, as shown in Fig. 7B and C. After treatment of 4 weeks, rats of the MTX/ARPL@MS group showed much reduced redness and swelling of the ankle and phalangeal joints, and the activity of the rats was also improved compared to other groups. The average clinical score value of rats in the MTX/ARPL@MS group was 2.7 ± 0.6, which also better than the model (4.7 ± 0.6), ARS@MS (4.3 ± 0.6), and MTX + ARS@MS groups (3.3 ± 0.6). After measurement, the average paw thickness of rats in the MTX/ARPL@MS group was 9.9 ± 0.3 mm, which was much lower than that of the model group (12.4 ± 0.9 mm) and slightly lower than those of the ARS@MS (11.1 ± 0.7 mm) and the MTX + ARS@MS (10.9 ± 1.4 mm) groups. After treatment of 6 weeks, paw swelling of rats in the MTX/ARPL@MS group was reduced markedly and was quite close to the situation of rats in the healthy control group. Moreover, the joint lesion degree of rats in the MTX + ARS@MS group was also improved markedly, which was significantly better than that in the saline and ARS@MS groups. After measurement, the paw thickness of rats in the MTX/ARPL@MS group was also the smallest among all experimental groups, which was 7.5 ± 0.4 mm. Accordingly, its clinical score was 1.0 ± 1.0 mm, which was also closest to that of healthy mice. In the other two treatment groups (ARS@MS and MTX + ARS@MS), the paw thickness and the clinical score of the rats also improved markedly. Meanwhile, the paw thickness and clinical score of the model group were even slightly improved compared with those at week 4, indicating that the disease of the AIA model mice may progress further without treatment. Therefore, through morphological observation and clinical score data, the therapeutic effect of intra-articular injection of MTX/ARPL@MS on the AIA model mice was preliminarily verified.

X-ray imaging study is also an important means to examine the pathological development of RA. Six weeks after administration, the metatarsophalangeal joints of rats in each group were detected by x-ray, due to the most marked RA-like symptoms presented in such surrounding small joints of the injection location. The images were shown in Fig. 7D. Compared with the healthy group, the soft tissue around the metatarsophalangeal joint of rats in the model group had obvious shadow (white boxes), decreased bone mineral density (white boxes), a small amount of hyperosteoplasia (yellow arrows), and joint capsule swelling and joint space narrowing (red arrows). The hyperplasia of bone may be related to the periosteum reaction caused by the adjuvant, with ectopic bone hypertrophy. Bone mineral density in the MTX/ARPL@MS group was similar to that in the healthy group, and osteoporosis was improved. In the ARS@MS group, bone mineral density was increased after treatment, but hyperosteoplasia still existed and joint space was reduced. In the MTX + ARS@MS group, bone mineral density was further increased without bone erosion or hyperplasia, and the joint space was slightly narrowed. The AIA model can well simulate the pathological process of RA, which mainly consists of synovial lesion and joint space change. In this experiment, joint inflammation was predominant in all groups without obvious bone erosion, which may be related to the unbalanced osteoclast remodeling mechanism of local immune response caused by complete adjuvant.

In addition, peripheral blood serum samples were retained from the orbit before the rats were sacrificed. Inflammatory cytokines including TNF-α and IL-1β exist not only in the synovium of the joint cavity but also in the peripheral circulating blood [31]. As shown in Fig. 7E and F, the TNF-α and IL-1β cytokine levels of in peripheral blood serum were detected by ELISA. After treatment by MTX/ARPL@MS, the TNF-α level was decreased to 0.18 ± 0.01 pg/ml, and IL-1β was also decreased to 0.21 ± 0.01 pg/ml, which was close to the levels of the healthy group, which were 0.16 ± 0.01 and 0.18 ± 0.00 pg/ml, respectively. Rats injected with ARS@MS or MTX + ARS@MS also showed decreased TNF-α and IL-1β expressions, which were still higher than the animals in the MTX/ARPL@MS group. ELISA data clearly showed that the intra-articular local treatment by MTX/ARPL@MS could also affect systemic inflammatory response through detecting the peripheral blood inflammatory cytokine level.

**Fig. 8.**
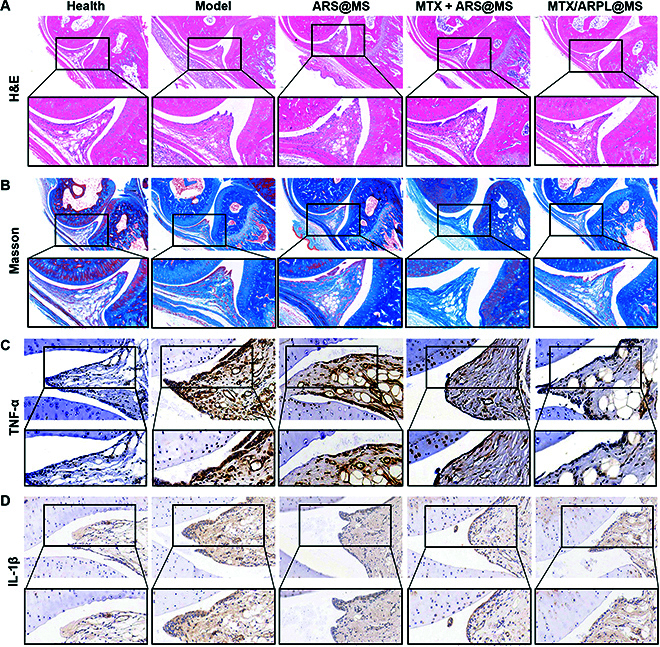
Histological analysis of in vivo assay. (A) Representative H&E staining images of metatarsophalangeal joints with partially enlarged details. (B) Representative Masson staining images of metatarsophalangeal joints with partially enlarged details. (C and D) Representative immunohistochemical images of metatarsophalangeal joints by detecting TNF-α (C) and IL-1β (D), with partially enlarged details.

After animals were sacrificed, the tissue samples, including hearts, livers, spleens, lungs, and kidneys, were fixed in paraformaldehyde and embedded in paraffin. The tissue sections after hematoxylin-eosin (H&E) staining were shown in Fig. 7G. Compared with the healthy group, no significant historical change occurred in all experimental groups. The data indicated that the local administration in ankle joints was quite safe. Then, we focused on synovial histopathology in phalangeal joints as these adjacent small joints showed marked reduction in symptoms. H&E, Masson, and immuohistochemical stains were conducted to joint sections. As shown in Fig. 8A, H&E staining showed that the synovial tissue in the healthy group was loose and porous, mainly the honeycomb adipose tissue, and there was little inflammatory cell infiltration. In the model group, the inflammatory reaction caused by complete adjuvant led to the proliferation of connective tissue, which replaced the original adipose tissue. Inflammatory cells were mostly distributed around the synovium, and small blood vessels were formed in some areas. After the MTX/ARPL@MS treatment, the degree of synovial inflammation was reduced, and connective tissue hyperplasia and inflammatory cell infiltration were greatly improved. As shown in Fig. 8B, Masson staining results also confirmed that the MTX/ARPL@MS group could achieve a better anti-inflammatory effect under intervention. Subsequently, the expression levels of TNF-α and IL-1β were detected by immunohistochemical staining. As shown in Fig. 8C and D and corresponding statistical analysis in Fig. S10, the content of inflammatory cytokines in the model group was much higher than that in the healthy group, which formed dark brown deposition. Both the ARS@MS group and the MTX + ARS@MG group showed different degrees of anti-inflammatory ability. MTX/ARPL@MS can strongly inhibit the aggregation of inflammatory cytokines, effectively preventing the progression of inflammation. The expression levels of TNF-α and IL-1β were different depending on the degree of inflammatory cascade induced by the adjuvant. In general, these data further confirmed the efficacy of local injected MTX/ARPL@MS for RA at the histological level.

We found that ARP can achieve effective nonenzyme-dependent activation in situ, which provides a possibility for its application in situ injection. Although the stability of ARP and its assembly (ARPL) is greatly improved compared with that of DHA active molecules, it is still not sufficient for local long-term administration. Therefore, we creatively used HAMA microsphere system to immobilize ARPL through reversible Schiff base imine structure to maintain its stability in long-term retention therapy. The obtained MTX/ARPL@MS nano/microsphere would be a better option for the in situ-activated therapy for localized diseases such as RA*.* The in vivo results further confirmed our hypothesis. Finally, we deeply believe that this phospholipid-mimic strategy also has the guiding significance for the remodeling local usage of other similar previously regarded systemic prodrugs to realize in situ-activated therapy for localized diseases. Moreover, considering the increasing discovered new indications of artemisinin-based drugs, ARPL-based in situ-activated therapy has a great potential to further expand the application in the fields including other autoimmune diseases, as well as viruses, tumors, and inflammation.

Finally, this phospholipid-mimic strategy may have a certain degree of universal application for some systemically administrated drugs for an improved in situ-activated therapy. Here, the in situ-activated therapy of phospholipid mimics comes from their natural features of prodrug strategy. Through modification of some pharmacophores with cleavable chemical bonds, the efficacy of active molecules would be suppressed temporarily. While entering some lesion sites with specific environment, prodrugs would experience designed chemical bond cleavage to be converted to the previous active molecules. Therefore, we deeply believe that this phospholipid-mimic strategy also has the guiding significance for the remodeling local usage of other systemic administrated prodrugs to realize the in situ-activated therapy for localized diseases.

## Materials and Methods

Materials and Methods could be seen in the Supplementary Materials.

## Conclusions

In this work, we first constructed a local activable nano/microsphere based on an artemisinin-medicated phospholipid mimic (ARP) and realized the in situ enzyme-independent activation and long-term efficacy of artemisinin-based prodrugs in the joint cavity for RA treatment for the first time. The ability of in situ conversion/activation of ARPL was preserved after sustained release. MTX was loaded with ARPL, which can achieve a “drug-in-drug” co-delivery to the same target cells and make use of better synergy. Therefore, MTX/ARPL@MS realized in situ conversion/activation of artemisinin drugs by local injection into the articular cavity for the first time, which solved the contradiction between efficacy and stability of artemisinin precursors. The specific formulation may provide a new option for RA in situ-activated therapy after preclinical evaluation.

## Data Availability

All data included in this study are available upon request by contact with the corresponding authors.

## Supplementary Materials

Supplementary Materials Supplementary Materials and Methods. Fig. S1. Synthesis of ARP. Fig. S2. ^1^H-NMR spectrum of ARP. Fig. S3. HRMS spectrum of ARP. Fig. S4. The HPLC elution curves of DHA and ARP after incubation in PBS for 3 and 24 h. Fig. S5. LS-MS/MS analysis, area vs. concentration scatter diagrams of DHA, ARS, and ARP. Fig. S6. The LC-MS/MS elution curves (MRM model) of ARS and ARP after incubation in PBS for 0 to 24 h. Fig. S7. Synthesis of DSPE-PEG-CN-SH. Fig. S8. ^1^H-NMR spectrum of DSPE-PEG-CN-SH. Fig. S9. CryoEM images of self-assembled ARPL without MTX loading. Fig. S10. Statistical analysis of immunohistochemical images.

## References

[1] Gao L, Yi M, Xing M, Li H, Zhou Y, Xu Q, Zhang Z, Wen Z, Chang J. *In situ* activated mesenchymal stem cells (MSCs) by bioactive hydrogels for myocardial infarction treatment. J Mater Chem B. 2020;8(34):7713–7722. 32724972 10.1039/d0tb01320j

[2] Huang K, Li F, Yuan K, Yang Y, Chang H, Liang Y, Yan X, Zhao J, Tang T, Yang S. A MOF-armored zinc-peroxide nanotheranostic platform for eradicating drug resistant bacteria via image-guided and *in situ* activated photodynamic therapy. Appl Mater Today. 2022;28:101513.

[3] Wang X, Li M, Hou Y, Li Y, Yao X, Xue C, Fei Y, Xiang Y, Cai K, Zhao Y, et al. Tumor-microenvironment-activated in situ self-assembly of sequentially responsive biopolymer for targeted photodynamic therapy. Adv Funct Mater. 2020;30(40):2000229.

[4] Ren C, Liu H, Lv F, Zhao W, Gao S, Yang X, Jin Y, Tan Y, Zhang J, Liang XJ, et al. Prodrug-based nanoreactors with tumor-specific *in situ* activation for multisynergistic cancer therapy. ACS Appl Mater Interfaces. 2020;12(31):34667–34677. 32610896 10.1021/acsami.0c09489

[5] Pu Y, Zhou B, Xiang H, Wu W, Yin H, Yue W, Yin Y, Li H, Chen Y, Xu H. Tyrosinase-activated prodrug nanomedicine as oxidative stress amplifier for melanoma-specific treatment. Biomaterials. 2020;259:120329. 32836058 10.1016/j.biomaterials.2020.120329

[6] Wei L, Chen J, Ding J. Sequentially stimuli-responsive anticancer nanomedicines. Nanomedicine (Lond). 2021;16(4):261–264. 33543644 10.2217/nnm-2021-0019

[7] Lukacova V, Goelzer P, Reddy M, Greig G, Reigner B, Parrott N. A physiologically based pharmacokinetic model for ganciclovir and its prodrug valganciclovir in adults and children. AAPS J. 2016;18(6):1453–1463. 27450227 10.1208/s12248-016-9956-4

[8] Watson DJ, Laing L, Gibhard L, Wong HN, Haynes RK, Wiesner L. Toward new transmission-blocking combination therapies: Pharmacokinetics of 10-amino-artemisinins and 11-aza-artemisinin and comparison with dihydroartemisinin and artemether. Antimicrob Agents Chemother. 2021;65(8):Article e0099021. 34097488 10.1128/AAC.00990-21 PMC8284440

[9] Chen L, Zheng Z, Liu H, Wang X, Qu S, Yang Y, Deng S, Zhang Y, Tuo L, Zhao Y, et al. Combined transcriptome and proteome profiling for role of pfEMP1 in antimalarial mechanism of action of dihydroartemisinin. Microbiol Spectr. 2021;9(3):Article e0127821. 34908430 10.1128/Spectrum.01278-21 PMC8672878

[10] Laleve A, Panozzo C, Kühl I, Bourand-Plantefol A, Ostojic J, Sissoko A, Tribouillard-Tanvier D, Cornu D, Burg A, Meunier B, et al. Artemisinin and its derivatives target mitochondrial c-type cytochromes in yeast and human cells. Biochim Biophys Acta Mol Cell Res. 2020;1867(5):118661. 10.1016/j.bbamcr.2020.118661 31987792

[11] Svensson US, Ashton M. Identification of the human cytochrome P450 enzymes involved in the in vitro metabolism of artemisinin. Br J Clin Pharmacol. 1999;48(4):528–535. 10583023 10.1046/j.1365-2125.1999.00044.x PMC2014388

[12] Tiwari MK, Chaudhary S. Artemisinin-derived antimalarial endoperoxides from bench-side to bed-side: Chronological advancements and future challenges. Med Res Rev. 2020;40(4):1220–1275. 31930540 10.1002/med.21657

[13] Zhang Y, Xu G, Zhang S, Wang D, Saravana Prabha P, Zuo Z. Antitumor research on artemisinin and its bioactive derivatives. Nat Prod Bioprospect. 2018;8(4):303–319. 29633188 10.1007/s13659-018-0162-1 PMC6102173

[14] Yang FM, Fan D, Yang XQ, Zhu FH, Shao MJ, Li Q, Liu YT, Lin ZM, Cao SQ, Tang W, et al. The artemisinin analog SM934 alleviates dry eye disease in rodent models by regulating TLR4/NF-κB/NLRP3 signaling. Acta Pharmacol Sin. 2021;42(4):593–603. 32747720 10.1038/s41401-020-0484-5 PMC8114933

[15] Su T, Feng X, Yang J, Xu W, Liu T, Zhang M, Ding J, Chen X. Polymer nanotherapeutics to correct autoimmunity. J Control Release. 2022;343:152–174. 34990701 10.1016/j.jconrel.2021.12.036

[16] Abbasi M, Mousavi MJ, Jamalzehi S, Alimohammadi R, Bezvan MH, Mohammadi H, Aslani S. Strategies toward rheumatoid arthritis therapy; the old and the new. J Cell Physiol. 2019;234(7):10018–10031. 30536757 10.1002/jcp.27860

[17] Lee JS, Oh JS, Kim YG, Lee CK, Yoo B, Hong S. Methotrexate-related toxicity in patients with rheumatoid arthritis and renal dysfunction. Rheumatol Int. 2020;40(5):765–770. 32170389 10.1007/s00296-020-04547-y

[18] Zhou CX, Li L, Ma YG, Li BN, Li G, Zhou Z, Shi F, Weng J, Zhang C, Wang F, et al. A bioactive implant in situ and long-term releases combined drugs for treatment of osteoarticular tuberculosis. Biomaterials. 2018;176:50–59. 29857274 10.1016/j.biomaterials.2018.05.039

[19] Lei Y, Wang Y, Shen J, Cai Z, Zhao C, Chen H, Luo X, Hu N, Cui W, Huang W. Injectable hydrogel microspheres with self-renewable hydration layers alleviate osteoarthritis. Sci Adv. 2022;8(5):Article eabl6449. 35108047 10.1126/sciadv.abl6449 PMC8809544

[20] Chen Z, Zhang F, Zhang H, Cheng L, Chen K, Shen J, Qi J, Deng L, He C, Santos HA, et al. DNA-grafted hyaluronic acid system with enhanced injectability and biostability for photo-controlled osteoarthritis gene therapy. Adv Sci. 2021;8(9):2004793. 10.1002/advs.202004793 PMC8097319 33977074

[21] Lei Y, Wang X, Liao J, Shen J, Li Y, Cai Z, Hu N, Luo X, Cui W, Huang W. Shear-responsive boundary-lubricated hydrogels attenuate osteoarthritis. Bioact Mater. 2022;16:472–484. 35415286 10.1016/j.bioactmat.2022.02.016 PMC8967971

[22] Lin F, Wang Z, Xiang L, Deng L, Cui W. Charge-guided micro/nano-hydrogel microsphere for penetrating cartilage matrix. Adv Funct Mater. 2021;31(49):2107678.

[23] Ismail M, Ling L, Du Y, Yao C, Li X. Liposomes of dimeric artesunate phospholipid: A combination of dimerization and self-assembly to combat malaria. Biomaterials. 2018;163:76–87. 29454237 10.1016/j.biomaterials.2018.02.026

[24] Du Y, Giannangelo C, He W, Shami GJ, Zhou W, Yang T, Creek DJ, Dogovski C, Li X, Tilley L. Dimeric artesunate glycerophosphocholine conjugate nano-assemblies as slow-release antimalarials to overcome Kelch 13 mutant artemisinin resistance. Antimicrob Agents Chemother. 2022;66(5):Article e0206521. 35416709 10.1128/aac.02065-21 PMC9112877

[25] Adebayo JO, Tijjani H, Adegunloye AP, Ishola AA, Balogun EA, Malomo SO. Enhancing the antimalarial activity of artesunate. Parasitol Res. 2020;119(9):2749–2764. 32638101 10.1007/s00436-020-06786-1 PMC7340003

[26] Tang J, Xi K, Chen H, Wang L, Li D, Xu Y, Xin T, Wu L, Zhou Y, Bian J, et al. Flexible osteogenic glue as an all-in-one solution to assist fracture fixation and healing. Adv Funct Mater. 2021;31(38):2102465.

[27] Sun Y, Zhou Q, Du Y, Sun J, Bi W, Liu W, Li R, Wu X, Yang F, Song L, et al. Dual biosignal-functional injectable microspheres for remodeling osteogenic microenvironment. Small. 2022;18(36):Article e2201656. 35419952 10.1002/smll.202201656

[28] Han Y, Pang X, Pi G. Biomimetic and bioinspired intervention strategies for the treatment of rheumatoid arthritis. Adv Funct Mater. 2021;31(38):2104640.

[29] Yang Y, Guo L, Wang Z, Liu P, Liu X, Ding J, Zhou W. Targeted silver nanoparticles for rheumatoid arthritis therapy via macrophage apoptosis and re-polarization. Biomaterials. 2021;264:120390. 32980634 10.1016/j.biomaterials.2020.120390

[30] He W, Du Y, Wang T, Wang J, Cheng L, Li X. Dimeric artesunate–phosphatidylcholine-based liposomes for irinotecan delivery as a combination therapy approach. Mol Pharm. 2021;18(10):3862–3870. 34470216 10.1021/acs.molpharmaceut.1c00500

[31] Zhang Y, He W, Du Y, Du Y, Zhao C, Zhang Y, Zhang H, Yin L, Li X. Dimeric artesunate phospholipid-conjugated liposomes as promising anti-inflammatory therapy for rheumatoid arthritis. Int J Pharm. 2020;579:119178. 32105722 10.1016/j.ijpharm.2020.119178

[32] Feng X, Liu J, Xu W, Li G, Ding J. Tackling autoimmunity with nanomedicines. Nanomedicine (Lond). 2020;15(16):1585–1597. 32669025 10.2217/nnm-2020-0102

[33] Feng X, Xu W, Li Z, Song W, Ding J, Chen X. Immunomodulatory nanosystems. Adv Sci. 2019;6(17):1900101. 10.1002/advs.201900101 PMC6724480 31508270

[34] Du Y, He W, Xia Q, Zhou W, Yao C, Li X. Thioether phosphatidylcholine liposomes: A novel ROS-responsive platform for drug delivery. ACS Appl Mater Interfaces. 2019;11(41):37411–37420. 31556583 10.1021/acsami.9b08901

[35] Gabriel NE, Roberts MF. Spontaneous formation of stable unilamellar vesicles. Biochemistry. 1984;23(18):4011–4015. 6487587 10.1021/bi00313a001

[36] Harris JK, Rose GD, Bruening ML. Spontaneous generation of multilamellar vesicles from ethylene oxide/butylene oxide diblock copolymers. Langmuir. 2002;18:5337–5342.

[37] Maus A, Strait L, Zhu D. Nanoparticles as delivery vehicles for antiviral therapeutic drugs. Eng Regen. 2021;2:31–46. 38620592 10.1016/j.engreg.2021.03.001 PMC7988306

[38] Wang T, Bai J, Lu M, Huang C, Geng D, Chen G, Wang L, Qi J, Cui W, Deng L. Engineering immunomodulatory and osteoinductive implant surfaces via mussel adhesion-mediated ion coordination and molecular clicking. Nat Commun. 2022;13(1):160. 35013289 10.1038/s41467-021-27816-1 PMC8748715

[39] Du Y, Wang Z, Wang T, He W, Zhou W, Li M, Yao C, Li X. Improved antitumor activity of novel redox-responsive paclitaxel-encapsulated liposomes based on disulfide phosphatidylcholine. Mol Pharm. 2020;17(1):262–273. 31747284 10.1021/acs.molpharmaceut.9b00988

[40] Luo Z, Che J, Sun L, Yang L, Zu Y, Wang H, Zhao Y. Microfluidic electrospray photo-crosslinkable κ-Carrageenan microparticles for wound healing. Eng Regen. 2021;2:257–262.

[41] Chen Z, Lv Z, Zhang Z, Zhang Y, Cui W. Biomaterials for microfluidic technology. Materials Futures. 2022;1(1):Article 012401.

[42] Cai Z, Saiding Q, Cheng L, Zhang L, Wang Z, Wang F, Chen X, Chen G, Deng L, Cui W. Capturing dynamic biological signals *via* bio-mimicking hydrogel for precise remodeling of soft tissue. Bioact Mater. 2021;6(12):4506–4516. 34027237 10.1016/j.bioactmat.2021.04.039 PMC8134719

[43] Yang J, Han Y, Lin J, Zhu Y, Wang F, Deng L, Zhang H, Xu X, Cui W. Ball-bearing-inspired polyampholyte-modified microspheres as bio-lubricants attenuate osteoarthritis. Small. 2020;16(44):Article e2004519. 32940012 10.1002/smll.202004519

[44] Genovese RF, Newman DB. Understanding artemisinin-induced brainstem neurotoxicity. Arch Toxicol. 2008;82(6):379–385. 17972063 10.1007/s00204-007-0252-z

[45] Dovedytis M, Liu ZJ, Bartlett S. Hyaluronic acid and its biomedical applications: A review. Eng Regen. 2020;1:102–113.

[46] Zhao D, Zhang J, Xu G, Wang Q. Artesunate protects LPS-induced acute lung injury by inhibiting TLR4 expression and inducing Nrf2 activation. Inflammation. 2017;40(3):798–805. 28315999 10.1007/s10753-017-0524-6

[47] Gremese E, Alivernini S, Tolusso B, Zeidler MP, Ferraccioli G. JAK inhibition by methotrexate (and csDMARDs) may explain clinical efficacy as monotherapy and combination therapy. J Leukoc Biol. 2019;106(5):1063–1068. 31313387 10.1002/JLB.5RU0519-145R PMC6852123

[48] Ma JD, Jing J, Wang JW, Yan T, Li QH, Mo YQ, Zheng DH, Gao JL, Nguyen KA, Dai L. A novel function of artesunate on inhibiting migration and invasion of fibroblast-like synoviocytes from rheumatoid arthritis patients. Arthritis Res Ther. 2019;21(1):153. 31234900 10.1186/s13075-019-1935-6 PMC6591920

[49] Yao W, Wang F, Wang H. Immunomodulation of artemisinin and its derivatives. Sci Bull. 2016;61:1399–1406.

[50] Hu HM, Mao MH, Hu YH, Zhou XC, Li S, Chen CF, Li CN, Yuan QL, Li W. Artemisinin protects DPSC from hypoxia and TNF-α mediated osteogenesis impairments through CA9 and Wnt signaling pathway. Life Sci. 2021;277:119471. 33811898 10.1016/j.lfs.2021.119471

[51] Huang X, Xie Z, Liu F, Han C, Zhang D, Wang D, Bao X, Sun J, Wen C, Fan Y. Dihydroartemisinin inhibits activation of the Toll-like receptor 4 signaling pathway and production of type I interferon in spleen cells from lupus-prone MRL/lpr mice. Int Immunopharmacol. 2014;22(1):266–272. 25027631 10.1016/j.intimp.2014.07.001

[52] Zhang T, Zhang X, Lin C, Wu S, Wang F, Wang H, Wang Y, Peng Y, Hutchinson MR, Li H, et al. Artemisinin inhibits TLR4 signaling by targeting co-receptor MD2 in microglial BV-2 cells and prevents lipopolysaccharide-induced blood-brain barrier leakage in mice. J Neurochem. 2021;157(3):611–623. 33453127 10.1111/jnc.15302

[53] Tu J, Hong W, Zhang P, Wang X, Körner H, Wei W. Ontology and function of fibroblast-like and macrophage-like synoviocytes: How do they talk to each other and can they be targeted for rheumatoid arthritis therapy? Front Immunol. 2018;9:1467. 29997624 10.3389/fimmu.2018.01467 PMC6028561

[54] Du H, Zhang X, Zeng Y, Huang X, Chen H, Wang S, Wu J, Li Q, Zhu W, Li H, et al. A novel phytochemical, DIM, inhibits proliferation, migration, invasion and TNF-α induced inflammatory cytokine production of synovial fibroblasts from rheumatoid arthritis patients by targeting MAPK and AKT/mTOR signal pathway. Front Immunol. 2019;10:1620. 31396207 10.3389/fimmu.2019.01620 PMC6663984

[55] Efferth T, Oesch F. The immunosuppressive activity of artemisinin-type drugs towards inflammatory and autoimmune diseases. Med Res Rev. 2021;41(6):3023–3061. 34288018 10.1002/med.21842

[56] Sun Y, Yao Y, Ding CZ. A combination of sinomenine and methotrexate reduces joint damage of collagen induced arthritis in rats by modulating osteoclast-related cytokines. Int Immunopharmacol. 2014;18(1):135–141. 24287449 10.1016/j.intimp.2013.11.014

[57] Umar S, Palasiewicz K, Volin MV, Romay B, Rahat R, Tetali C, Arami S, Guma M, Ascoli C, Sweiss N, et al. Metabolic regulation of RA macrophages is distinct from RA fibroblasts and blockade of glycolysis alleviates inflammatory phenotype in both cell types. Cell Mol Life Sci. 2021;78(23):7693–7707. 34705053 10.1007/s00018-021-03978-5 PMC8739866

[58] Chen F, Jiang G, Liu H, Li Z, Pei Y, Wang H, Pan H, Cui H, Long J, Wang J, et al. Melatonin alleviates intervertebral disc degeneration by disrupting the IL-1β/NF-κB-NLRP3 inflammasome positive feedback loop. Bone Res. 2020;8:Article 10. 10.1038/s41413-020-0087-2 PMC7028926 32133213

[59] Zampeli E, Vlachoyiannopoulos PG, Tzioufas AG. Treatment of rheumatoid arthritis: Unraveling the conundrum. J Autoimmun. 2015;65:1–18. 26515757 10.1016/j.jaut.2015.10.003

[60] Maudens P, Seemayer CA, Pfefferlé F, Jordan O, Allémann E. Nanocrystals of a potent p38 MAPK inhibitor embedded in microparticles: Therapeutic effects in inflammatory and mechanistic murine models of osteoarthritis. J Control Release. 2018;276:102–112. 29524442 10.1016/j.jconrel.2018.03.007

[61] Scott DL, Wolfe F, Huizinga TW. Rheumatoid arthritis. Lancet. 2010;376(9746):1094–1108. 20870100 10.1016/S0140-6736(10)60826-4

