# Supplementary material for: In Situ-Activated Phospholipid-Mimic Artemisinin Prodrug via Injectable Hydrogel Nano/Microsphere for Rheumatoid Arthritis Therapy
Source: Research (Wash D C). 2022 Dec 15;2022:0003. doi: 10.34133/research.0003 (PMC11407526; doi:10.34133/research.0003)
Supplement: Supplementary Materials — and Methods. Fig. S1. Synthesis of ARP. Fig. S2. 1H-NMR spectrum of ARP. Fig. S3. HRMS spectrum of ARP. Fig. S4. The HPLC elution curves of DHA and ARP after incubation in PBS for 3 and 24 h. Fig. S5. LS-MS/MS analysis, area vs. concentration scatter diagrams of DHA, ARS, and ARP. Fig. S6. The LC-MS/MS elution curves (MRM model) of ARS and ARP after incubation in PBS for 0 to 24 h. Fig. S7. Synthesis of DSPE-PEG-CN-SH. Fig. S8. 1H-NMR spectrum of DSPE-PEG-CN-SH. Fig. S9. CryoEM images of self-assembled ARPL without MTX loading. Fig. S10. Statistical analysis of immunohistochemical images. [file 0003.f1.docx]

**Supplementary information**

***In situ* activated** **phospholipid-mimic artemisinin prodrug *via* injectable hydrogel** **nano/microsphere for rheumatoid arthritis** **therapy**

Yawei Du ^1,#^, Chao Li ^1,2,#^, Yu Zhang ^1^, Wei Xiong ^1^, Fei Wang ^1^, Juan Wang ^1^, Yingze Zhang ^2^, Lianfu Deng ^1^, Xinsong Li ^3,*^, Wei Chen ^2,*^, Wenguo Cui ^1,*^

1. Department of Orthopaedics, Shanghai Key Laboratory for Prevention and Treatment of Bone and Joint Diseases, Shanghai Institute of Traumatology and Orthopaedics, Ruijin Hospital, Shanghai Jiao Tong University School of Medicine, 197 Ruijin 2nd Road, Shanghai 200025, P. R. China.

2. Department of Orthopaedic Surgery, the Third Hospital of Hebei Medical University, No.139 Ziqiang Road, Shijiazhuang 050051, P. R. China.

3. School of Chemistry and Chemical Engineering, Southeast University, 2 Southeast University Road, Nanjing 211189, P. R. China.

# These authors contributed equally to this work.

* Corresponding authors: lixs@seu.edu.cn (X. Li), surgeonchenwei@126.com (W. Chen), wgcui80@hotmail.com (W. Cui)

**Supplementary Content**

**S1 Materials and methodsS2**

S1.1 MaterialsS2

S1.2 Synthesis of ARPS2

S1.3 Synthesis of DSPE-PEG-CN-SH and DSPE-PEG-HAP-1S2

S1.4 Preparation of MTX/ARPL S3

S1.5 Characterization of MTX/ARPLS3

S1.6 Preparation of nano/microsphere S4

S1.7 Characterization of nano/microsphereS5

S1.8 *In vitro* cytotoxicity assay S6

S1.9 *In vitro* anti-RA assay *via* analyzing inflammatory expression of M1-type macrophages S6

S1.10 *In vitro* anti-RA assay *via* analyzing migration/invasion of RA-FLSS7

S1.11 *In vitro* anti-RA assay *via* M1-type macrophages and RA-FLS co-cultivationS8

S1.12 *In vivo* anti-RA assayS8

S1.13 Statistical analysisS9

**S2 Supplementary figuresS10**

**ReferencesS13**

**S1 Materials and methods**

**S1.1 Materials**

Artesunate (ARS), N,N’-carbonyldiimidazole (CDI), 1,8-diazabicyclo-(5.4.0)-undec-7-ene (DBU), cysteamine and DSPE-PEG-NHS were purchased from Aladdin Co. (Shanghai, China). L-α glycerylphosphorylcholine (GPC) was purchased from ZZBIO Co., Ltd. (Shanghai, China). DSPE-PEG-CHO was purchased from Ponsure Biotech. Co. (Shanghai, China). HAP-1 peptide was purchased from Qiangyao BioTech. Co. (Wuhan, China). DSPE-PEG-FA was purchased from Pengsheng BioTech. Co. (Shanghai, China). Methotrexate (MTX) and cholesterol were purchased from J&K Scientific Co. (Shanghai, China). Sodium hyaluronate (HA) was purchased from Bloomage Biotech. Co. (Beijing, China). Lipopolysaccharides (LPS) and interferon-γ (IFN-γ) were purchased from Beyotime Biotech. Co. (Nantong, China). RAW264.7 cell line was purchased from National Collection of Authenticated Cell Culture (Shanghai, China). MH7A cell line was gifted by Prof. Peng Zhang from Shenzhen Institutes of Advanced Technology, Chinese Academy of Sciences (Shenzhen, China). All the solvents used were purchased from Sinopharm Co. (Shanghai, China).

**S1.2 Synthesis of ARP**

ARP was synthesized by ARS and GPC through an esterification reaction which was catalyzed by CDI/DBU system as reported previously(*1*). Briefly, ARS (0.128 g) was firstly activated by CDI (0.243 g) in DMSO (10 mL) solvent for 2 h at RT. After that, GPC (0.026 g) and DBU (0.05 g) were dissolved in DMSO (5 mL) and then added to the reaction. And the mixture was stirred at RT for another 12 h. Finally, the reaction mixture was separated directly by a SiO_2_ column with gradient elution of dichloromethane/methanol (5/1) and dichloromethane/methanol/water (65/25/4).

**S1.3 Synthesis of DSPE-PEG-CN-SH and DSPE-PEG-HAP-1**

Then, DSPE-PEG-CN-SH was synthesized by DSPE-PEG-CHO and cysteamine through a Schiff base reaction (As shown in Figure S7). Briefly, DSPE-PEG-CHO and cysteamine (molar ratio, 1:5) were dissolved in DMSO, and stirred at 40 ^o^C with catalytic amount of p-toluene sulfonic acid for overnight. Subsequently, the mixture was purified by dialysis (MWCO 1500). After lyophilization, the DSPE-PEG-CN-SH powder was obtained.

DSPE-PEG-HAP-1 was synthesized by DSPE-PEG-NHS and HAP-1 peptide through an amidation reaction. Briefly, DSPE-PEG-NHS and HAP-1 peptide (molar ratio, 1:5) were dissolved in DMSO/water (1/4) solvent with the pH value adjusted to around 8.5. Keep stirring for 2 h at RT. Then, the mixture was purified by Sephadex G50 chromatography. After lyophilization, the DSPE-PEG-HAP-1 powder was obtained.

**S1.4 Preparation of MTX/ARPL**

MTX/ARPL liposomes were prepared by a classic thin-film dispersion method(*1*). Briefly, ARP, cholesterol, MTX, DSPE-PEG-FA, DSPE-PEG-HAP-1 and DSPE-PEG-CN-SH (molar ratio, 60:20:10:4:4:2) were dissolved in 20 mL of dichloromethane in a 250 mL round-bottom flask. The solvent was evaporated under [reduced pressure](javascript:;) at 40 ^o^C to form a lipid thin film. After that, appropriate PBS buffer (pH 7.4) was added to hydrate the thin film at 40 ^o^C for 30 min. The liposomal suspension was further homogenized by a Mini-Extruder (610000 Avanti Polar Lipids) for at least 10 times. And the blank ARPL without MTX was prepared by the similar method except adding MTX.

**S1.5 Characterization of MTX/ARPL**

The morphology of blank ARPL and MTX/ARPL was observed by CryoEM. 4 μL of liposomal suspension (lipid concentration of 5 mg/mL) was added on 300-mesh grids. The cryo-samples were made by Vitrobot Mark IV (ThermoFisher, Hillsboro, OR). The Blot time, blot force and wait time were set as 4 s, -1 and 30 s, respectively. After that, samples were imaged by a field-emission transmission electron microscope (Tecnai G2 200 kV, FEI, Hillsboro, OR). The size distribution was measured using a Zetasizer Nano ZS90 Instrument (Malvern Instruments Ltd. Worcestershire, UK). 1 mL of MTX/ARPL suspension (lipid concentration of 1 mg/mL) was added to sample cells and set the scattering angle as 173^o^.

The drug release behavior of MTX and DHA from MTX/ARPL were detected by a dialysis method. Briefly, 5 mL of MTX/ARPL suspension (lipid concentration of 20 mg/mL) was added to a dialysis bag (MWCO 8000). Then, the dialysis bag was immersed into 500 mL of simulated body fluid with 0.5% Tween 80 and incubated at 37 °C. After different time intervals, 1 mL of external buffer solution was extracted, and 1 mL of simulated body fluid was re-added. The released MTX and DHA were measured by a spectrophotometer (EV300, ThermoFisher, Hillsboro, OR) and LC-MS (Dionex Ultimate 3000 UHPLC, ThermoFisher, Hillsboro, OR), respectively.

The cellular uptake behavior of MTX/ARPL was studied by flow cytometry. Two ligands (FA and HAP-1 peptide) were applied for the surface modification of MTX/ARPL to target inflammatory macrophage and synovial fibroblast according to the literatures (*2, 3*). And DSPE-PEG-FITC was added in the liposomal formulation to label MTX/ARPL with fluorescence signal. Briefly, RAW 264.7 and MH7A were seeded in 24-well plates and incubated for 12 h, while LPS and IFN-γ was added to final concentration of 1 μg/mL and 50 ng/mL respectively, to induce M0-type macrophages to M1-type. Then FITC labelled MTX/ARPL with/without FA and HAP-1 ligands were added to the mixture with finally lipid concentration of 1 μg/mL. After incubation at 37 ^o^C for 1 h. Cells were fixed by paraformaldehyde for 30 min. Finally, the cell suspension samples were tested by a CytoFLEX Flow Cytometer (Beckman Coulter Co., Brea, CA).

The lysosome escape assay of MTX/ARPL was studied by laser scanning confocal microscope. Briefly, LPS-induced RAW 264.7 and MH7A cells were seeded in confocal dishes for 12 h. Then, FITC-labelled MTX/ARPL was added to get the final lipid concentration of 1 μM. After incubation of 0.5 and 4 h, the cells were fixed. Besides, the Lysotracker-Red (Beyotime Biotechnology Co., Nantong, China), a lysosomal dye, was added before the cell fixation and incubated with cells for at least 0.5 h. Finally, the fixed cell samples were observed by laser scanning confocal microscope (Carl Zeiss Ltd., Oberkochen, Germany).

**S1.6 Preparation of nano/microsphere**

HAMA was synthesized by HA and methacrylic anhydride by a reported method [40]. Briefly, 10 g of HA (MW 400,000) was dissolved in 500 mL of DI water under mechanical stirring. Then, 20 mL of methacrylic anhydride was added slowly to the mixture followed by adding 20 mL of 5 M NaOH. Keep stirring on ice for 12 h. Finally, HAMA was obtained after dialysis (MWCO 8000) and lyophilization.

MTX/ARPL@MS nano/microspheres were prepared by a microfluidic instrument. Briefly, 1.5g (5%) of Span-80 was dissolved in 30 g (95%) of paraffin oil as the oil phase. 0.2 wt.% of MTX/ARPL, 0.25 wt.% of photoinitiator LAP and 2 wt.% of HAMA dissolved in DI water as the aqueous phase. Then, both oil and aqueous phase solution were filled in different syringes fixed in the microfluidic pump. Adjust the appreciate flow rate to formulate microspheres with different size distribution. A plate was placed on the fluid level of a cold trap (<-15^o^C) to collect the cut aqueous liquid globules (microspheres). After that, the frozen nanoliposome embedded microspheres (nano/microspheres) were irradiated under 365 nm UV for 15 min. Photo-induced crosslink within HAMA and thiol-ene click reaction between thiol-modified MTX/ARPL and HAMA were happened simultaneously. The nano/microspheres were washed by ether to remove paraffin oil. Finally, the nano/microsphere powder was obtained after lyophilization.

**S1.7 Characterization of nano/microsphere**

The morphology of MTX/ARPL@MS was observed under optical microscope before and after lyophilization. At the same time, the size of microspheres was measured from the photos by ImageJ software. To measure the swelling rate of lyophilized MTX/ARPL@MS powder after immersed in aqueous phase. 3 mg of MTX/ARPL@MS powder was added into 1.5 mL tube with 1 mL of PBS buffer (pH 7.4) for 3 h with 30 min intervals. At each time points, the supernate was removed and the residual water was absorbed by filter paper before weighting. The swelling rate was calculated according to the [wet weight](javascript:;) values. After DiR labelling, the distribution of MTX/ARPL within microsphere was observed by fluorescence microscope. The preparation of DiR labelled MTX/ARPL@MS were similar with the original microspheres, except adding DiR during the MTX/ARPL preparation process. After lyophilization, the chemical constitution of blank MS and MTX/ARPL@MS were investigated by ATR-FTIR. The morphology and EDS were investigated by a high-resolution field-emission scanning electron microscopy (Sirion 200, FEI, Hillsboro, OR).

The liposomal release rate was detected by centrifugal method. Briefly, 10 mg of MTX/ARPL@MS powder was added into 15 mL centrifuge tube with 10 mL of simulated body fluid for different time intervals. At each time points, centrifuge at 1500 rpm for 2 min, and 1 mL of buffer was collected, and 1 mL of fresh simulated body fluid was re-added. The UV spectrum was obtained by a spectrophotometer, and the amount of released liposomes was calculated measured by a spectrophotometer (EV300, ThermoFisher, Hillsboro, OR) according to the standard curve.

Besides, the degradation situation was carried out by optical microscope as well. To observe the degradation of MTX/ARPL@MS, nano/microsphere powder (30 mg) was suspended in 1 mL of simulated body fluid containing hyaluronidase (1500 U/mL). Samples were incubated at 37 ℃ for 2 months. Simulated body fluid was renewed by fresh liquid every three days. The destroy of MTX/ARPL@MS was observed per week under the optical microscope.

**S1.8** ***In vitro* cytotoxicity assay**

The *in vitro* cytotoxicity of MTX/ARPL@MS was evaluated by cell live/dead staining *via* Calcein-AM/PI and viability assay *via* CCK8. For cell live/dead staining, MH7A cells were seeded in 24-well plate with density of 1×10^5^ cells/well and incubated at 37℃ for overnight. Then, the medium of each well was replaced with the medium containing extract of MTX@MG or MTX/dAPC@MG, which was extracted after 24 h incubation at 37℃. After incubation at 37℃ for 1 and 2 days, the cells were double-stained by Calcein-AM/PI and observed by a fluorescence microscope.

For CCK8 assay, three cell lines, including RAW264.7, MH7A and L929, were used to further investigate the cytotoxicity of MTX/ARPL@MS. Briefly, cells were seeded in 96-well plates with density of 1×10^4^ cells/well and incubated at 37℃ for overnight. Then, the medium of each well was replaced with the medium containing extract of MTX@MG or MTX/dAPC@MG, which was extracted after 24 h incubation at 37℃. After incubation at 37℃ for 1, 2 and 3 days, the cells were incubated with medium containing 10% CCK8 for 1 h. Finally, the plates were read by a microplate reader (ThermoFisher, Hillsboro, OR) at wavelength of 250 nm.

**S1.9 *In vitro* anti-RA assay *via* analyzing inflammatory expression of** **M1-type macrophages**

The anti-RA efficacy against M1-type macrophages of MTX/ARPL was investigated by detecting the expression of inflammatory cytokines *via* qPCR technique. Firstly, RAW264.7 cells were seeded in 6-well plates with density of 2×10^6^ cells/well and incubated at 37℃ for overnight. After that, the medium of each well was replaced with the medium containing ARS, MTX, MTX+ARS or MTX/ARPL (with MTX concentration of 80 nM or ARS equivalent concentration of 1 μM). M0-type RAW264.7 cells without induction and LPS-induced cells without treatment were used as controls. After incubation at 37℃ for 24 h, cells in each well were lysed by 0.5 mL of TRIzol and then mixed with 0.4 mL of CHCl_3_ by vortex mixer for 15 s. Samples were incubated at RT for 15 min. After centrifugation (12000 rpm, 4^o^C, 15 min), the supernate was collected and mixed with 1 mL of isopropanol for precipitation. After incubation at RT for 10 min, the tubes were centrifuged (12000 rpm, 4^o^C, 15 min) to obtain the RNA precipitation. The precipitation was washed by ethanol, and then dissolved by 20 μL of DEPC water.

The total RNA concentration of each sample was measured by a NanoDrop spectrophotometer (ThermoFisher, Hillsboro, OR). Then, reverse transcription was carried out by a PrimeScript kit (TaKaRa, Tokyo, Japan). Finally, qPCR was conducted by a SYBR Premix EX Taq II Kit (TaKaRa, Tokyo, Japan) on Thermo 7500 Instrument (ThermoFisher, Cleveland, OH). The mRNA expression was calculated by the 2^-ddCt^ method. Primer sequences: β-actin (forward: 5′-GGCTGTATTCCCCTCCATCG-3′, reverse: 5′-CCAGTTGGTAACAATGCCATGT-3′), TNF-α (forward: 5′-CCCTCACACTCAGATCATCTTCT-3′, reverse: 5′-GCTACGACGTGGGCTACAG-3′) β-actin (forward: 5′-GCAACTGTTCCTGAACTCAACT-3′, reverse: 5′- ATCTTTTGGGGTCCGTCAACT-3′).

**S1.10 *In vitro* anti-RA assay *via* analyzing migration/invasion of RA-FLS**

The expression of MMP2 in human RA-FLS (MH7A) stimulated by different drugs was firstly detected by Western blot. Briefly, MH7A cells were seeded in 6-well plates with density of 1×10^6^ cells/well and incubated at 37℃ for overnight. After that, the medium of each well was replaced with the medium containing ARS, MTX, MTX+ARS or MTX/ARPL (with MTX concentration of 80 nM or ARS equivalent concentration of 1 μM). After incubation at 37℃ for 24 h, cells in each well were lysed by 0.5 mL of cell lysates. After mixed with loading buffer, samples were added to different channels of SDS-Page gel and separated under electrophoresis. Then, the blots were transferred to the nitrocellulose membrane. The membrane was blocked with PBS buffer containing 3% of skim milk and 0.1% of Tween-20 (mPBST) for 1 h at RT. And then membrane was incubated with anti-MMP2 and anti-GAPDH primary antibodies at 4 ^o^C for overnight. After that, secondary antibodies were incubated with membrane for 1 h at RT. After enhanced chemiluminescent (ECL) treatment, the blots were imaged under the imaging system (Bio-Rad, Hercules, CA).

The migration inhibition effect of MTX/ARPL on MH7A cells was evaluated by cell scratch wound healing assay. Briefly, MH7A cells were seeded in 6-well plates with density of 5×10^5^ cells/well and incubated at 37℃ for overnight. After that, scratch the cells using 20 μL pipette tips carefully. Wash the plates by PBS buffer for 3 time, and add serum-free medium containing ARS, MTX, MTX+ARS or MTX/ARPL (with MTX concentration of 80 nM or ARS equivalent concentration of 1 μM). After incubation for 12 and 24 h, the scratch wounds were observed under an optical microscope.

Transwell technique was used to further evaluate the migration/invasion inhibition of MTX/ARPL@MS. The pore size of the filter membrane was 8 μm. For invasion assay, Matrigel (8-fold dilution) was used to coat the membrane for 30 min at 37^o^C. Before seeding, the MH7A cells were incubated with serum-free medium for 12 h. Then, cells were seeded on polyester membrane with density of 4×10^5^ cells/well and incubated at 37℃ for 24 h. At the same time, ARS@MS, MTX@MS, MTX+ARS@MS or MTX/ARPL@MS (with final MTX concentration of 800 nM or ARS equivalent concentration of 10 μM) were added the medium in the bottom of wells. Subsequently, the membranes were washed by PBS buffer for 2 time, and cells was fixed by methanol for 30 min followed by stained by trypan blue for 10 min. After washed by PBS buffer for 3 times, the cells on the underside of the membrane were observed by an optical microscope.

**S1.11 *In vitro* anti-RA assay *via* M1-type macrophages and RA-FLS co-cultivation**

Synovial macrophages (MLS) and synovial fibroblasts (FLS) are two prominent cellular components in synovial tissue. The M1-type macrophages/RA-FLS co-cultivation Transwell assay was designed, and the secreted TNF-α and IL-1β from M1-type macrophages and RA-FLS as well as the migration and invasion of RA-FLS were detected simultaneously. The pore size of the filter membrane was 8 μm. For invasion assay, Matrigel (8-fold dilution) was used to coat the membrane for 30 min at 37^o^C. RAW264.7 cells were seeded on the bottom of wells with density of 4×10^5^ cells/well and induced by medium containing LPS (1 μg/mL) and IFN-γ (50 ng/mL) at 37℃ for 24 h. Then, MH7A cells were seeded on polyester membrane with density of 4×10^5^ cells/well. After that, fresh medium containing ARS@MS, MTX@MS, MTX+ARS@MS or MTX/ARPL@MS (with final MTX concentration of 800 nM or ARS equivalent concentration of 10 μM) were added to the bottom of wells. After incubation at 37℃ for 24 h, the medium in each well was collected for detection of IL-1β and TNF-α by ELISA (Servicebio Biotech Co., Wuhan, China). At the same time, the membranes were washed by PBS buffer for 2 time, and cells was fixed by methanol for 30 min followed by stained by trypan blue for 10 min. After washed by PBS buffer for 3 times, the cells on the underside of the membrane were observed by an optical microscope.

**S1.12 *In vivo* anti-RA assay**

The animal experiment was approved by the Animal Ethics Committee of the Third Hospital of Hebei Medical University (K2021-012-1). 25 Sprague-Dawley (SD) rats (female, 8 weeks old) were purchased from JieSiJie Animals Co. (Shanghai. China). The rats were maintained in a specific pathogen free (SPF) grade experimental animal room. For AIA model establishment, 100 μL of freund's complete adjuvant (FCA) (1 mg/mL) was injected into the paw of rats, and another injection of 50 μL FCA was carried out 5 days later to intensive the immunization. The rats were divided into 5 groups randomly (n=5), including Health control, Model control, ARS@MS, MTX+ARS@MS and MTX/ARPL@MS groups. 2 weeks after modeling, 50 μL of injections including saline, ARS@MS, MTX+ARS@MS and MTX/ARPL@MS (MTX concentration of 80 μM or ARS equivalent concentration of 1 mM) started to be administrated by intra-articular injection into ankle joints. The injection was applied once a week.

After injection for 4 and 6 weeks, photos of paws were taken. And the general evaluation was made with a clinical score and the paw thickness was measured as well every two weeks. The clinical scores were evaluated based on the guidance as follows: the activity is normal and no symptoms (score 0); symptom is mild with slight diffuse erythema, and the paw is slightly swelling with one or both toes affected (score 1); symptom is mild, and the paw is swelling with 3-4 toes affected (score 2); symptom is moderate with slight diffuse erythema, and the entire paw is swelling (score 3); symptom is significant with obvious diffuse erythema, and the entire paw and ankle are swelling (score 4); symptom is severe, and entire paw and fingers are disabled (score 5).

X-ray imaging study is also an important means to examine the pathological development of RA. 6 weeks after administration, metatarsophalangeal joints of rats in each group were detected by X-ray (Faxitron X-ray Corporation, Buffalo Grove, IL). In addition, peripheral blood serum samples were retained from the orbit before the rats were sacrificed. Inflammatory cytokines including TNF-α and IL-1β were measured by ELISA (Servicebio Biotech Co., Wuhan, China). Besides, the tissue samples, including hearts, livers, spleens, lungs and kidneys, were fixed in paraformaldehyde, and embedded in paraffin, and stained by H&E. The metatarsophalangeal joints were collected. After de-calcification, the samples and embedded in paraffin. Then, standard H&E, Masson and immunohistochemical staining were conducted. The sections were observed under optical microscope.

**S1.13 Statistical analysis**

Data were shown as mean ± standard deviation (SD). Statistical comparison was performed by Student’s t-test and the significant difference were considered when *P* < 0.05.

**S2 Supplementary figures**

**Figure S1.** Synthesis of ARP.


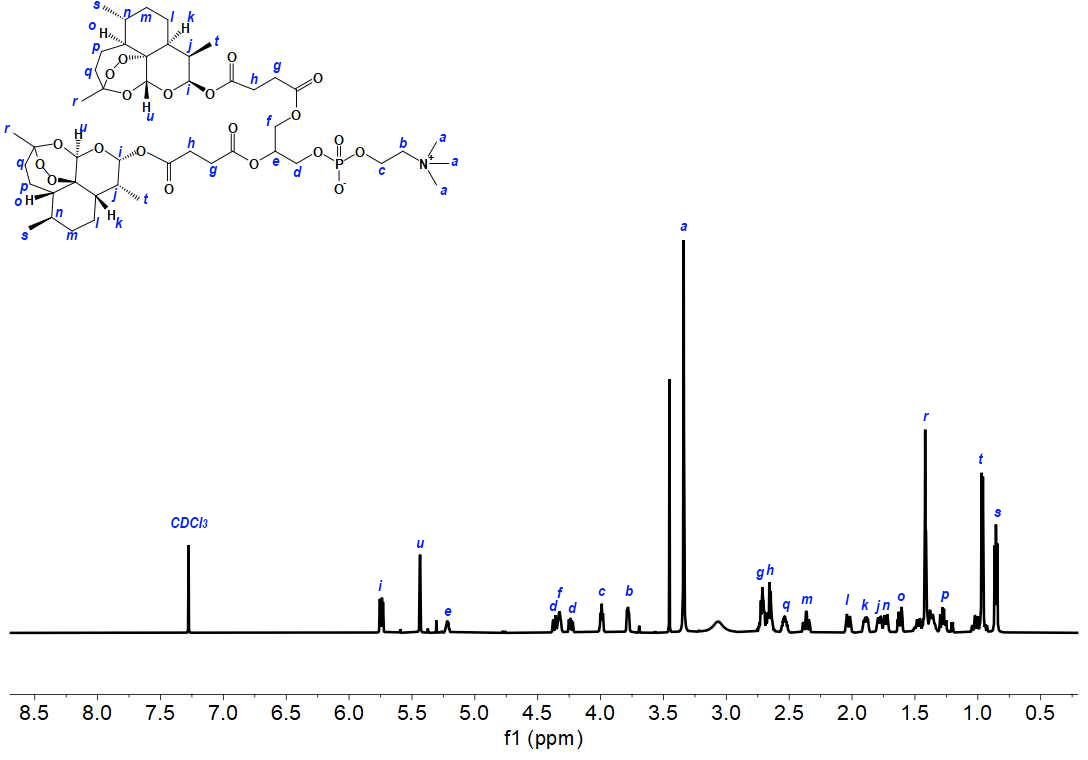


**Figure S2.** ^1^H-NMR spectrum of ARP.


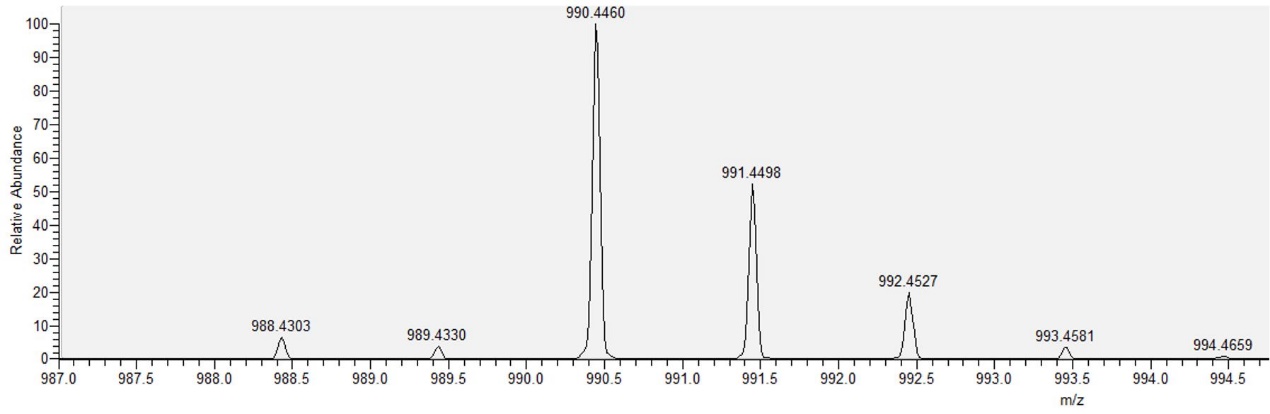


**Figure S3.** HRMS spectrum of ARP.


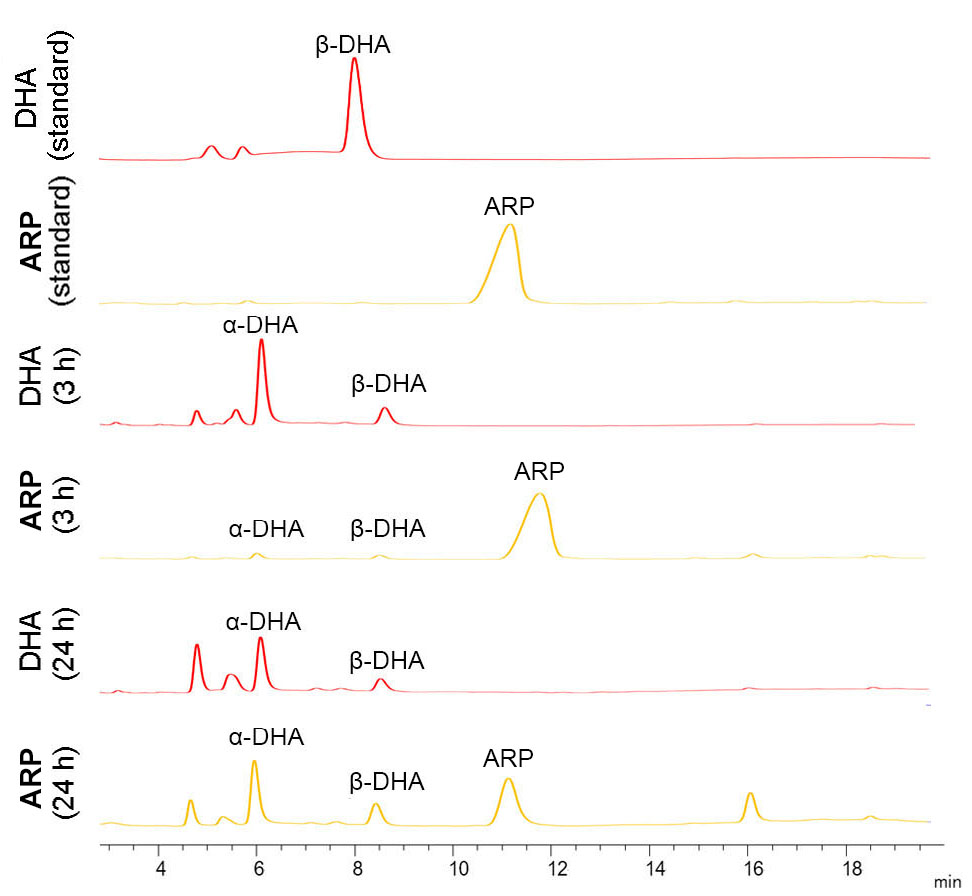


**Figure S4.** The HPLC elution curves of DHA and ARP after incubation in PBS for 3 and 24 h.


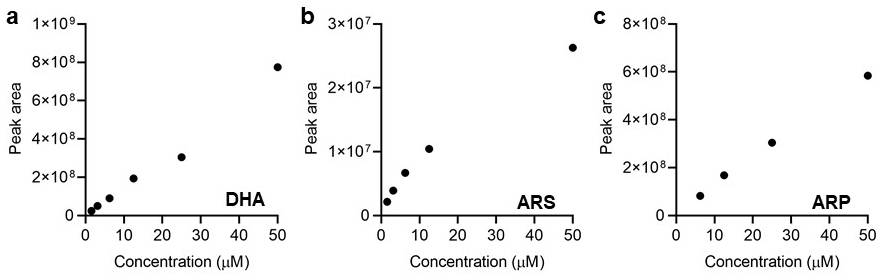


**Figure S5.** LS-MS/MS analysis, area *vs.* concentration [scatter diagram](javascript:;)s of DHA, ARS and ARP.


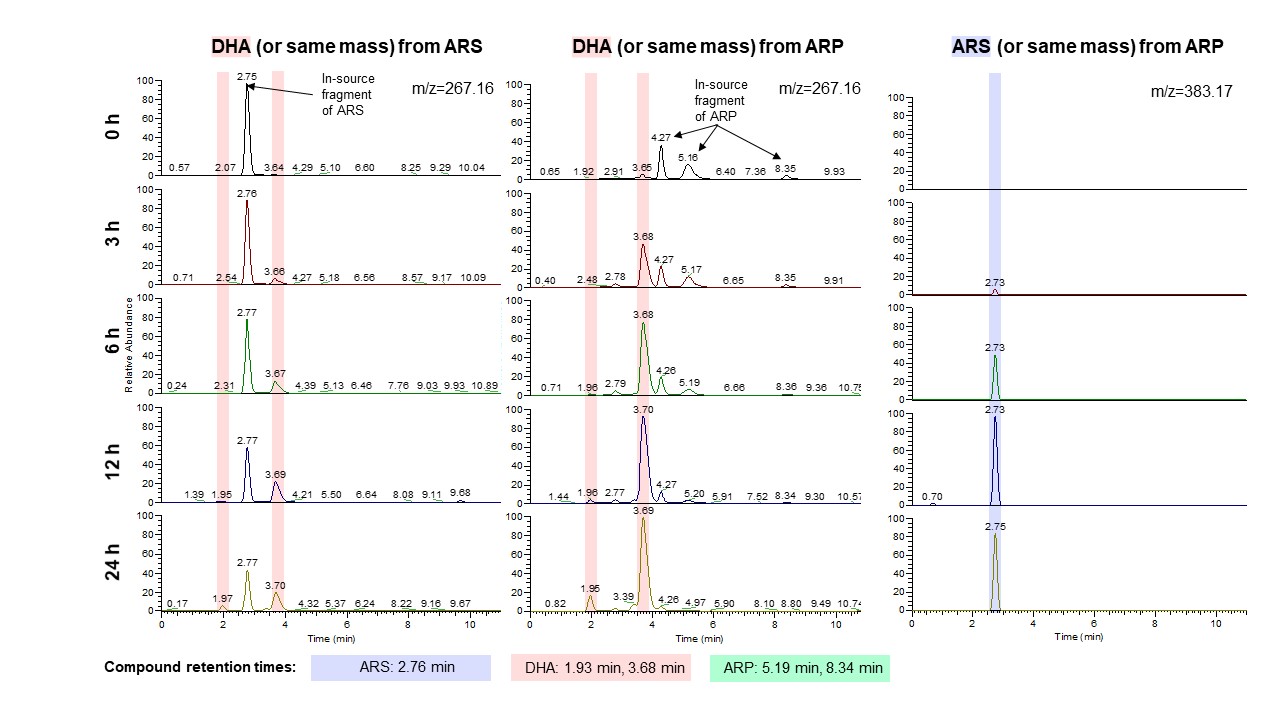


**Figure S6.** The LC-MS/MS elution curves (MRM model) of ARS and ARP after incubation in PBS for 0-24 h.

**Figure S7**. Synthesis of DSPE-PEG-CN-SH.


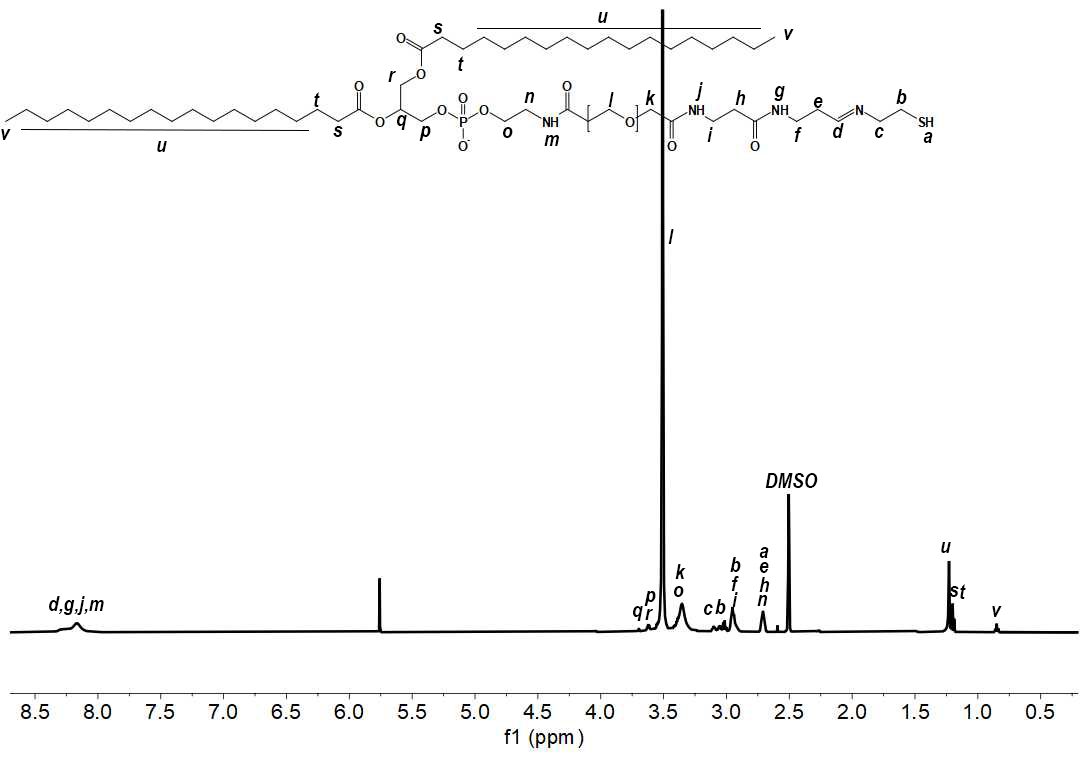


**Figure S8.** ^1^H-NMR spectrum of DSPE-PEG-CN-SH.


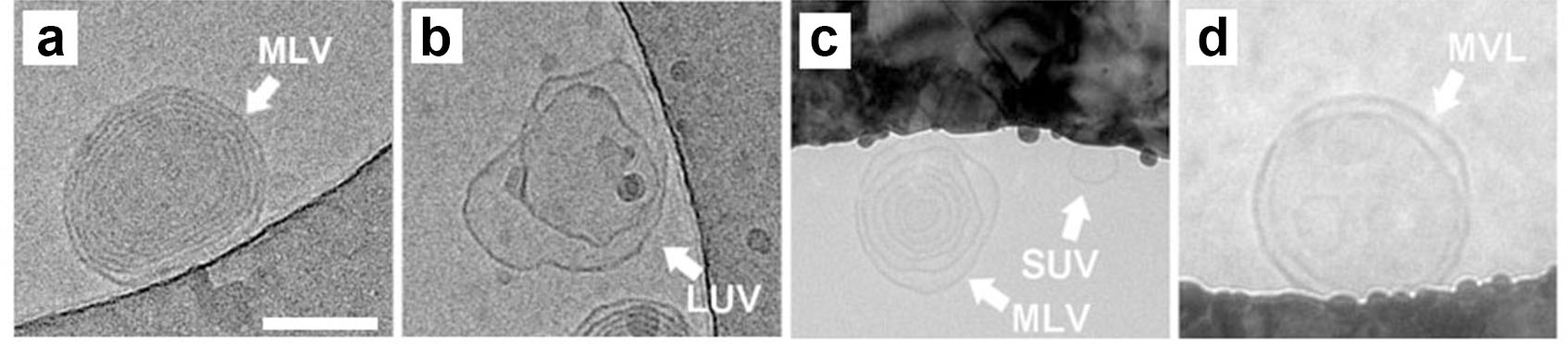


**Figure S9.** CryoEM images of self-assembled ARPL without MTX loading clearly presented liposomal vesicae with different structure, including single unilamellar vesicles (SUV), large unilamellar vesicles (LUV), multilamellar vesicles (MLV) and multivesicular liposomes (MVL).


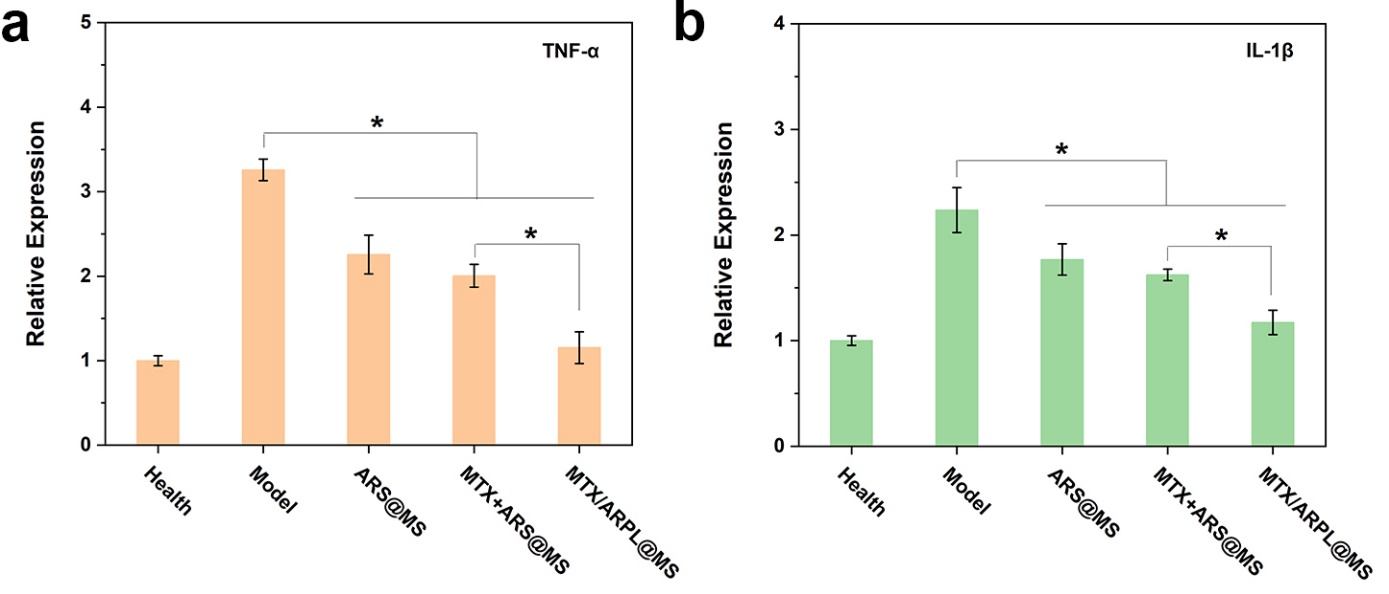


**Figure S10.** Statistical analysis of immunohistochemical images (Figure 7c and 7d)

**Reference**

1. M. Ismail, L. Ling, Y. Du, C. Yao, X. Li, Liposomes of dimeric artesunate phospholipid: A combination of dimerization and self-assembly to combat malaria. *Biomaterials* **163**, 76-87 (2018).

2. Y. Han, X. Pang, G. Pi, Biomimetic and Bioinspired Intervention Strategies for the Treatment of Rheumatoid Arthritis. *Advanced Functional Materials* **31**, 2104640 (2021).

3. Y. Yang *et al.*, Targeted silver nanoparticles for rheumatoid arthritis therapy via macrophage apoptosis and Re-polarization. *Biomaterials* **264**, 120390 (2021).
